# Decoding Smell from Receptor Structure

**DOI:** 10.21203/rs.3.rs-9947738/v1

**Published:** 2026-06-14

**Authors:** Hsiu-Yi Lu, Aashutosh Vihani, Maira H. Nagai, Xiaoyang S. Hu, Dan Takase, Conan Juan, Claire A. deMarch, Hiroaki Matsunami

**Affiliations:** 1 Department of Molecular Genetics and Microbiology, Duke University School of Medicine, Research Drive, Durham, NC 27710 USA; 2 Department of Neurobiology, Neurobiology graduate program, Duke University Medical Center, Durham, NC 27710, USA; 3 Department of Neurobiology, Duke University School of Medicine, Research Drive, Durham, NC 27710 USA

## Abstract

Olfaction enables animals to detect and discriminate an immense diversity of volatile chemicals, yet how individual odorant receptors determine ligand selectivity remains poorly understood (1–4). Recent cryo-electron microscopy structures of mammalian odorant receptors(5–11), together with advances in protein structure prediction(12–14), now provide an opportunity to test whether receptor structure can explain odor recognition at scale. Here, we combine AlphaFold3-predicted odorant receptor structures with ESM2-derived protein sequence embeddings and large-scale sequencing linked to in vivo olfactory sensory neuron activation across a chemically diverse odorant panel. Using these data, we develop a structure-informed deep learning framework that predicts odorant receptor-ligand interactions and maps relationships between receptor space and chemical space. The learned receptor representation organizes odorant receptors by functional similarity rather than primary sequence alone, while feature attribution identifies binding-cavity subregions that contribute to chemical recognition. Together, these results show that odorant receptor ligand selectivity is encoded in three-dimensional receptor features and establish a predictive framework for interpreting odorant receptor coding.

## Introduction

Animals rely on multiple sensory systems to navigate and interpret their environment, each specialized to encode distinct types of physical or chemical information. Among these, olfaction presents one of the least understood and most complex encoding challenges. In color vision, the task is to discriminate between wavelengths of light and is achieved through the tuning of a small number of cone opsins with distinct spectral sensitivities, which are a subfamily of class A G protein-coupled receptors (GPCRs), is central to how humans and other animals perceive light as distinct colors (15, 16). Odorant receptors (ORs) in vertebrates are also class A GPCRs; however, in contrast to vision, olfaction must distinguish among diverse chemical molecules, requiring large repertoires of odorant receptors (ORs). ORs ranging from hundreds to thousands to encode this high-dimensional chemical space, which constitute the largest subfamily of class A GPCRs (1), with humans expressing approximately 400 intact ORs and mice over 1,000 (3, 17, 18). Mammals devote a substantial fraction of their genomes to ORs, to navigate the environment (4, 19), which reflects the evolutionary importance of chemical sensing across species. Despite their central role in odor perception, a fundamental unresolved question is how an individual OR determines which chemicals it binds and how it responds across chemical space. Addressing this question is essential for understanding the logic by which chemical information is encoded at the receptor level, yet progress has been limited by long-standing technical barriers to obtaining high-resolution OR structures.

Recent cryo-EM studies have resolved structures of mammalian ORs, first with OR51E2 in complex with propionate (5–11). Notably, these cryo-EM structures revealed that odorants primarily occupy a conserved binding cavity location, suggesting a canonical structural locus for odorant recognition across ORs (5, 6, 20). Functional studies further demonstrate that mutations altering the shape or chemical properties of this cavity can shift ligand preference, suggesting ligand selectivity is primarily influenced by the geometry and physicochemical features of the binding pocket.

In parallel, advances in computational biology have transformed access to protein structural and evolutionary information. Deep learning-based structure prediction methods such as AlphaFold3 (12, 13, 21), have provided the potential for accurate *in silico* predictions for the entire OR repertoire, offering a complementary resource where experimental structures are lacking. Additionally, other approaches like protein large language models (LLMs), such as the Evolutionary Scale Model (ESM) (22) extend this progress by embedding sequence-based evolutionary information into learned vector spaces that infer functional and biophysical constraints. Together, these approaches overcome a major limitation by providing receptor-level structural information at scale, creating an opportunity to pursue structure-informed modeling of OR-ligand interactions.

Many existing efforts to model olfactory perception and OR-ligand interactions have focused on ligand descriptors, psychophysical measurements, and receptor sequence features, yielding important insights into odor coding and chemical space (23–30). These approaches offer interpretable representations of odorant chemistry and perceptual outcomes. However, the limited availability of high-resolution receptor structures has limited the ability to connect ligand binding to three-dimensional features of the receptor binding cavity. Given that the receptor structure plays a central role in determining ligand specificity and activation (5, 6), integrating structural information is essential for directly linking chemical recognition to receptor function.

To address this gap, we developed an end-to-end modeling framework that integrates AlphaFold-predicted OR structures with ESM2-derived sequence embeddings within convolutional neural networks to predict OR-ligand interactions. Central to this effort is the use of pS6-IP-Seq, an in vivo assay that captures activation of olfactory sensory neurons through enrichment of phosphorylated ribosomal protein S6-associated transcripts (31, 32). While numerous studies have reported OR-ligand interactions, these datasets are typically small, heterogeneous in experimental design, and difficult to integrate across studies. In contrast, collection of pS6-IP-Seq studies enables systematic, parallel measurement of receptor activation across chemically diverse odorants within a unified experimental framework, making it particularly well suited for machine learning applications.

By integrating large-scale pS6-IP-Seq activation data with predicted receptor structures and sequence-derived embeddings, we set out to ask whether receptor structure representation can be explicitly leveraged to model and interpret OR-ligand interactions. Specifically, we aimed to determine the structural features of the receptor that encodes for the ligand selectivity and receptor tuning. Through this approach, we seek to establish a structure-informed framework for linking receptor feature to odorant activation, and to assess whether focusing on a canonical binding cavity can provide a mechanistic basis for understanding olfactory coding at the receptor level.

## Results

### pS6-IP-Seq as a Strategy to Identify Odorant Activated OR In Vivo

**Figure 1.**
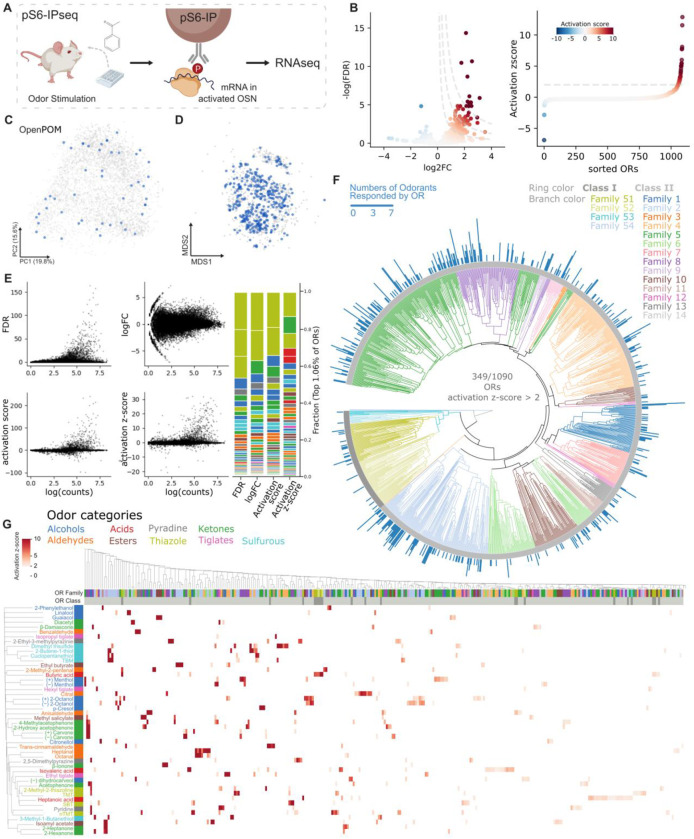
Defining odor-evoked olfactory receptor activation using pS6-IP **A**. Schematic of the pS6-IP-Seq experimental workflow. Odor exposure induces phosphorylation of ribosomal protein S6 in activated olfactory sensory neurons (OSNs). pS6-IP-Seq enriches mRNAs expressed in activated OSNs, which are subsequently profiled by RNA sequencing to identify odor-responsive olfactory receptors (ORs). **B**. Quantification of OR activation in response to 100% acetophenone stimulation. Left: Volcano plot showing enrichment of OR mRNAs, with points colored by activation score. Right: ORs ranked by activation z-score, with colors indicating activation score. The horizontal dashed line denotes the activation z-score threshold (> 2) used to define activated receptors. **C**. Chemical perceptual space representations of tested odorants. Principal component analysis (PCA) of 4680 small molecules based on OpenPOM. The 48 odorants tested by pS6-IP, each activating at least one OR, are highlighted in blue. **D**. ORs space representations of responding receptors. Multidimensional scaling (MDS) visualization of intact ORs based on pairwise Grantham distances. ORs responding to at least one of the 48 odorants are shown in blue (n = 349). **E**. Scatter plots of different metric’s (FDR, logFC, activation score, activation z-score) relationship biases with receptor counts. Right: Histogram showing the fraction of identified top 1% odor-ligand pairs selected using different response metrics. **F**. Circular phylogenetic tree of ORs, with branch colors indicating OR family and shaded ranges denoting OR class. The outer ring displays a bar plot representing the number of odorants to which each OR responds, as defined by activation z-score threshold > 2. **G**. Heatmap summarizing activation z-scores across all 48 pS6-IP odor experiments with activation z-score threshold > 2. Rows represent odorants and columns represent ORs, with hierarchical clustering applied to both dimensions.

The phosphorylation of the ribosomal protein S6 is a well-established marker of neuronal activity (33). Similar to the induction of immediate early genes such as c-Fos, pS6 reflects recent neuronal activation, but with the added advantage of being physically associated with mRNA molecules. As a result, phosphorylated ribosomal S6 (pS6) complex can be immunoprecipitated and coupled to RNA sequencing, enabling a direct molecular readout of transcriptional profiles from activated olfactory sensory neurons (OSNs) following odor exposure (Fig. 1A). Given, each mature OSN expresses a single olfactory receptor gene (34–40), we can directly assign OR identities to individual OSNs. This organization allows pS6-IP-Seq to link odor-evoked neuronal activity to specific OR identities in vivo. Consistent with this framework, previous studies have demonstrated the utility of pS6-IP-Seq in inferring odorant-OR interactions in vivo freely behaving mice (31, 41, 42).

To ensure broad coverage of chemical space, we selected a panel of 48 odorants spanning diverse functional groups, molecular scaffolds, and physicochemical properties from panel of small molecules commonly found in foods and fragrances (43). In addition to maximizing global chemical diversity, the panel was intentionally designed to include closely related chemical pairs, such as enantiomers and homologous series differing by carbon chain length, enabling assessment of receptor sensitivity to subtle structural variations. Projection of these odorants into a reduced chemical feature space using Morgan fingerprint based chemical features (44), as well as clustering within an Open Principal Odor Map (OpenPOM) derived perceptual space (27, 45), generated from message passing neural networks trained on human odor perception, confirmed that the selected panel captures both structural and perceptual diversity across odorants (Fig. 1C and fig. S1B).

### Defining Activation Score to Quantify OR Responses

Traditional identification of activated olfactory receptors in pS6-IP experiments often repurposes differential expression criteria developed for gene-level analyses, such as false discovery rate (FDR) thresholds (e.g., FDR < 0.05) or minimum log fold-change (logFC > 1). While effective for detecting broadly regulated transcripts, these metrics are not optimized for classifying receptor activation and introduce systematic biases when applied to ORs. LogFC is strongly influenced by transcript abundance and sequencing depth: ORs with low counts can exhibit inflated fold changes due to small denominators, whereas highly expressed receptors frequently display more modest logFC values despite reproducible enrichment. As a result, logFC-based selection preferentially highlights sparsely expressed ORs while deprioritizing abundantly expressed receptors, confounding biological activation with sampling effects (Fig. 1E). In contrast, FDR-based selection introduces a complementary but distinct bias. Because FDR reflects statistical confidence rather than effect size, it preferentially prioritizes receptors with stable baseline expression and low variance, even when the magnitude of enrichment is small. Consequently, receptors exhibiting consistent but weak changes can be classified as activated, whereas biologically meaningful but variable responses may be excluded. Together, reliance on either logFC or FDR alone emphasizes different statistical regimes rather than receptor activation, limiting their interpretability for OR classification.

To address these limitations, we developed a composite metric, termed the Activation Score, which integrates both enrichment magnitude and statistical confidence without relying on a single criterion alone.

$$Activation\;Score=-\log(FDR)*logFoldChange\left(\frac{Odor}{No\;Odor}\right)$$

By jointly considering effect size and reliability, the Activation Score captures activation signals that reduces sensitivity to transcript abundance, variance structure, and arbitrary threshold selection. The advantage of this approach is illustrated in volcano plots of acetophenone pS6-IP responses (Fig. 1B), where increasing Activation Score thresholds yield a more balanced identification of responsive ORs compared with conventional FDR- or logFC-based filtering (Fig. 1E).

Furthermore, because different odorants elicit varying overall activation strengths at fixed experimental concentrations, direct comparison of raw activation scores across odors can introduce additional bias. To mitigate this, we normalized activation scores by z-scoring within each odor experiment, generating an odor-specific activation z-score. This normalization ensures that OR detection reflects relative responsiveness within an odor condition rather than absolute signal magnitude. Using this normalized metric, we defined activated ORs as those with an activation z-score greater than 2 for all downstream analyses.

Comparison of the top 1.06% of OR-odorant pairs, corresponding to the number of pairs exceeding an activation z-score of 2. Whereas FDR- and logFC-based criteria were skewed toward small subsets of odorants, activation z-score-based selection preserved a more diverse distribution of odor categories (Fig. 1E), indicating reduced bias toward ligands that produce exceptionally strong or abundant signals (46).

Analysis of OR responses identified using the activation z-score revealed broad coverage of receptor space. Projection of activated ORs into a sequence-based receptor space defined by Grantham distances demonstrated that activated receptors were widely distributed rather than concentrated within specific OR families or classes (Fig. 1D). This result was further supported by phylogenetic analysis, in which activated ORs were dispersed throughout the OR tree (Fig. 1F). Together, these analyses indicate that pS6-IP enables deorphanization across a diverse and representative fraction of the ORs.

Consistent with this diversity, clustering of OR-odorant responses across all 48 odor’s pS6-IPseq revealed that receptors did not group according to family identity, which is defined by overall amino acid similarities (47), but rather according to shared ligand responsiveness (Fig. 1G). Conversely, ORs with high sequence similarity did not necessarily respond to the same odorants, highlighting the dissociation between phylogenetic proximity and functional tuning.

Together, the odor-normalized Activation Score z-scored provide a more balanced and interpretable framework for quantifying OR activation from pS6-IP data. This metric reduces bias associated with transcript abundance and differential response strength, forming a robust foundation for the downstream structural modeling and machine learning analyses presented in this study.

### Encoding Odorant Receptor Structure

Having established a large-scale, chemically diverse dataset of in vivo OR activation, we next asked whether receptor structure contains information to predict ligand selectivity. Specifically, we sought to determine whether ORs could be represented in a structure-informed space that reflects their likelihood to respond to a given chemical stimuli, thereby enabling an interpretable model of receptor coding.

We hypothesized that the three-dimensional architecture of the receptor, particularly features within the ligand-binding cavity encodes constraints that shapes chemical recognition. Under this hypothesis, structural representations alone should organize ORs into a space that reflects their patterns of chemical responsiveness, even without incorporating ligand features.

**Figure 2.**
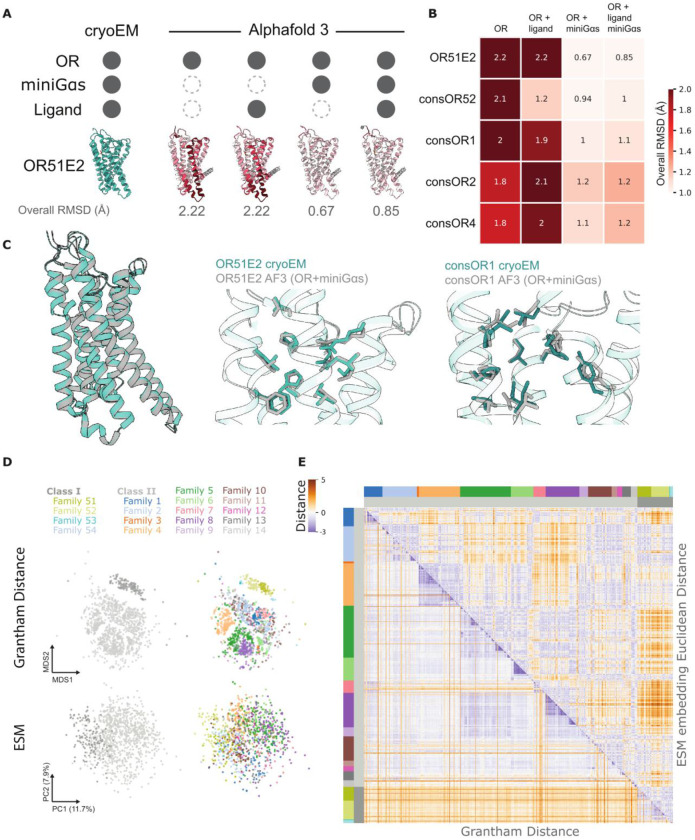
Structural accuracy of AlphaFold3 models and comparison of sequence- and language model-based receptor representations. **A**. Structural comparison between AlphaFold3-predicted models and the cryo-EM structure of OR51E2. Columns show different multimer configurations used for structure prediction. Structures are colored by per-residue RMSD relative to the cryo-EM reference, with overall RMSD values reported below each model. **B**. Heatmap summarizing overall RMSD values between AlphaFold3 predictions and experimentally determined cryo-EM structures across five available OR structures. **C**. Structural overlays of predicted and experimental OR structures. Left: superposition of OR51E2 cryo-EM and AlphaFold3 (OR+miniGαs) models. Middle: Magnified view of binding cavity residues for OR51E2, comparing cryo-EM and AlphaFold3 models. Right: Magnified view of binding cavity residues for consOR1, comparing cryo-EM and AlphaFold3 models. **D**. Low-dimensional visualization of OR similarity based on sequence-derived and language model-derived representations colored by class and family. Top: Multidimensional scaling (MDS) of pairwise Grantham distances. Bottom: Principal component analysis (PCA) of ESM-2 sequence embeddings. **E**. Pairwise distance heatmap showing Grantham distance based similarities (lower triangle) and Euclidean distances computed from ESM-2 embeddings (upper triangle) across ORs.

To establish a reliable structural foundation for this analysis, we first evaluated the ability of AlphaFold3 to generate accurate, activation-relevant OR conformations. As a test case, we modeled OR51E2 in complex with a mini Gαs protein using AlphaFold3 multimer. Notably, inclusion of the mini-Gαs protein alone was sufficient to stabilize an active-like receptor conformation, without providing the ligand (fig. S3). Comparison of the predicted structure with the recently solved cryo-EM structure of OR51E2 (5) revealed less than 1Å RMSD overall backbone deviation, indicating close agreement between predicted and experimental models (Fig. 2A and fig. S3). This result is consistent with prior observations that AlphaFold predictions tend toward inactive-like states when modeled in isolation but can be shifted toward active conformations through inclusion of signaling partners such as G proteins (Fig. 2A and B)(5). Importantly, similar structural concordance was observed across additional OR cryo-EM structures spanning distinct classes and subfamilies, with AlphaFold3 models showing consistently low backbone deviations. Together, these results support the use of AlphaFold3 with OR with miniGαs to generate active-like structural models for diverse ORs (Fig. 2B and fig. S3).

We next assessed whether these predicted structures were suitable for interrogating ligand selectivity by examining the architecture of the binding cavity Analysis of residues lining the predicted ligand-binding pocket showed that AlphaFold3 accurately recapitulates not only the overall receptor fold but also the positioning of key side chains that define cavity shape (Fig. 2C). The close correspondence between predicted and experimentally observed side-chain orientations indicates that AlphaFold3 models preserve the structural features critical for defining the binding pocket, supporting their use for downstream structural encoding and machine learning analyses.

### Feature encoding of primary sequence

To examine how sequence-derived representations capture OR similarity, we leveraged embeddings generated by the Evolutionary Scale Model (ESM), a protein language model trained on large-scale protein sequences to learn contextual and evolutionary patterns directly from sequence data (22). Unlike traditional representations based on primary amino acid identity or predefined substitution matrices, ESM embeddings encode each residue in the context of its surrounding sequence enabling the representation to capture higher-order relationships. ESM-based embeddings have been shown to correlate with protein structure, mutational effects, and functional similarity across diverse protein families (14, 48). To compare against a baseline measure of OR similarity from primary sequence, we computed pairwise distances using Grantham’s amino acid substitution matrix. These distances capture differences in physicochemical properties such as polarity, volume, and composition, and thus provide a familiar framework for comparing receptor families. Using multidimensional scaling, we projected the pairwise Grantham distances into two dimensions (Fig. 2D). The resulting clusters closely mirrored known OR class and family designations, confirming that physicochemical substitution metrics reflect evolutionary relationships across the OR repertoire as expected.

Full-length OR sequences were encoded using ESM, and the resulting embeddings were projected into principal component space for visualization (Fig. 2D). Compared with Grantham distance-based clustering, which primarily reflects amino acid similarity and substitution patterns, ESM embeddings showed weaker segregation by canonical OR families, suggesting that they capture sequence features beyond simple residue identity (Fig. 2D–E).

To directly compare these representations, we computed pairwise distances using both ESM embeddings and Grantham metrics. While the resulting distance matrices showed broad agreement at the level of OR class and family organization (Fig. 2E), they also revealed distinct patterns unique to each approach. Correlation analysis confirmed a moderate but incomplete relationship between the two measures (fig. S2), indicating that ESM embeddings encode additional evolutionary and contextual information not captured by traditional substitution-based metrics. Together, these results highlight that ESM provides a complementary, higher-order representation of OR sequence space.

### Integration of Structural Voxels and ESM Embeddings

**Figure 3.**
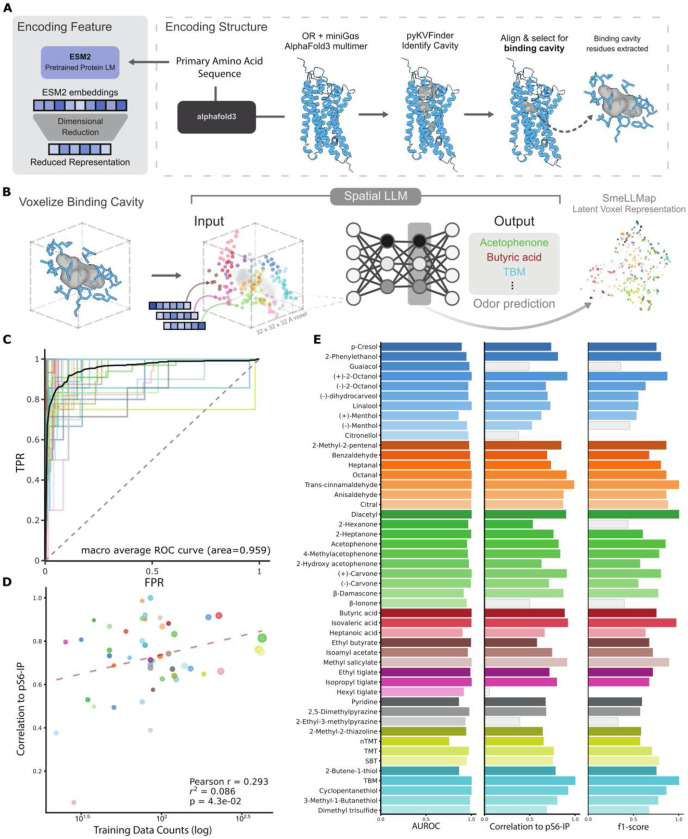
Spatial LLM architecture and model performance. **A**. Schematic of the data pre-processing pipeline. For sequence encoding, full-length OR sequences were input to ESM-2 to generate residue-wise embeddings, which were subsequently reduced in dimensionality by principal component analysis (PCA). In parallel, the same sequences were used to predict OR structures (OR+miniGαs) using AlphaFold3. Predicted structures were analyzed with pyKVFinder to identify internal cavities, which were filtered against predefined canonical binding cavity coordinates. Residues surrounding the retained binding cavity were extracted to generate a structural representation for each OR. **B**. Spatial LLM modeling pipeline. OR structural representations were voxelized into three-dimensional grids. Voxel occupancy encoded spatial structure, while reduced ESM-2 features were assigned to voxels based on residue identity, yielding a spatially and feature-aware representation of each OR. These voxel tensors were used as input to a convolutional neural network (CNN) trained to predict binary OR activation labels derived from pS6-IP activation z-scores. The output of the final fully connected layer was additionally extracted as a latent embedding, termed ‘smeLLMap’, representing learned receptor features. **C**. Receiver operating characteristic (ROC) curve summarizing CNN classification performance across all OR-odorant pairs with a macroaveraged curve plotted in black. **D**. CNN model prediction correlation to pS6-IP labels for individual odorants, plotted against the number of OR-ligand pairs in training data. Circle size is proportion to the number of test-set molecules. **E**. Model performance metrics evaluated per odorant, including area under the ROC curve (AUROC), correlation between prediction and pS6-IP activation values, and F1 score.

With validated structural models and residue-level embeddings in hand, we developed a unified spatial learning framework that integrates three-dimensional receptor architecture with sequence-derived features into a single representation (Fig. 3A). For each OR, the cavity space was calculated using pyKVFinder (49) from the AlphaFold3-predicted structure generated in complex with mini-Gα. These cavities were then filtered against the canonical binding cavity reference, yielding a single, standardized ligand-binding region for each receptor. Amino acid residues encasing this cavity space were extracted as the structural representation of the OR.

The resulting cavity-centered receptor structures were discretized into a three-dimensional voxel grid of size 32 x 32 x 32 at 1 Å resolution, with the grid centered on the predicted ligand-binding cavity (Fig. 3B). For each receptor, residues contributing to the binding cavity were first identified based on their spatial proximity, and each of their amino acid residue’s atomic coordinates were mapped onto the voxel grid according to their three-dimensional positions. Each occupied voxel was annotated with sequence-derived features obtained from ESM2 embeddings corresponding to the residue mapped to that spatial location. This voxelization strategy yields a spatially aligned, feature-aware representation of each receptor that jointly encodes binding cavity structure and residue identity for three-dimensional convolutional learning.

To provide functional supervision for training, we leveraged the pS6-IP-Seq data described above. Using the activation z-score metric greater than or equal to 2, which equates to roughly 0.05 of population in normal distribution, each OR was assigned to binary activated or inactive for each individually tested odors. This labeling scheme transforms the experimental response data into a multi-label classification task: given the structural and embedding representation of an OR, predict which odor it is likely to respond to. In this way, the pipeline effectively provides the first attempt at building an interpretable olfactory map directly from structural information.

### Convolutional Neural Network Odor Response Model

To predict OR odor responses, we trained a convolutional neural network (CNN) using voxelized binding cavity representations of 349 ORs responding to at least one odorant. For each receptor, five independent AlphaFold3 structural predictions were included, and voxel-level data augmentation was applied to increase robustness to minor structural variation. The dataset was randomly partitioned into training, validation, and test sets using a 70/15/15 split at the receptor level to prevent information leakage across folds. The model was optimized using the Adam optimizer with binary cross-entropy loss in a multi-label classification setting, reflecting the fact that individual receptors may respond to multiple odorants.

Across training runs, both training and validation losses decreased with no evidence of divergence between the two curves, indicating stable optimization without overfitting (fig. S4). Improvements in loss were accompanied by concordant increases in validation F1-score, demonstrating that optimization gains translated into improved classification performance rather than trivial fitting to dominant classes (fig. S4).

The final Spatial LLM CNN achieved consistently high predictive performance across the odor panel. Multi-class ROC analysis yielded a macro-averaged AUCROC of 0.959, indicating strong overall discriminative ability across odors (Fig. 3C). However, because the dataset is heavily class-imbalanced, with substantially more inactive than active OR-odorant pairs, ROC-AUC alone can overestimate performance due to the abundance of true negatives (50). To address this potential limitation, we evaluated additional performance metrics that are more sensitive to prediction confidence and class balance. First, we assessed whether model output probabilities reflected experimentally measured activation strength by correlating predicted confidence scores with pS6-IPseq activation values across receptors for each odorant (Fig. 3D). This analysis revealed strong correspondence between model confidence and biological activation, with 41 of 48 odorants exhibiting correlation coefficients greater than 0.6 and a mean correlation of 0.71 across odors (Fig. 3E and table S1). Second, precision-recall performance remained robust, with 32 of 48 odorants achieving F1-scores above 0.6 and a mean F1-score of 0.67, indicating balanced sensitivity and precision across most odor categories (Fig. 3D, fig. S4 and table S1).

We further examined the relationship between predictive performance and the number of positive training ORs available per odorant. Correlation strength increased with the number of activated ORs used for training, revealing a significant positive association between data availability and model performance (Fig. 3D). Notably, this trend was not deterministic, several odorants with relatively few positive examples nonetheless achieved high correlation and F1-scores, suggesting that certain odor response patterns are more easily inferred or generalized between receptors. Together, these complementary assessments confirm that the high ROC-AUC values reflect discriminative performance rather than artifacts of class imbalance. The Spatial LLM framework therefore provides an approach for predicting OR-odorant interactions from structural representations.

### Feature Embeddings

To interpret the learned representation of OR-odorant interactions, we extracted receptor embeddings from the final fully connected layer of the trained CNN. These vectors provide compact, high-level encodings of receptor structure after transformation through the convolutional architecture. Embeddings for each OR were retained alongside experimentally determined odor response labels for downstream analyses.

**Figure 4.**
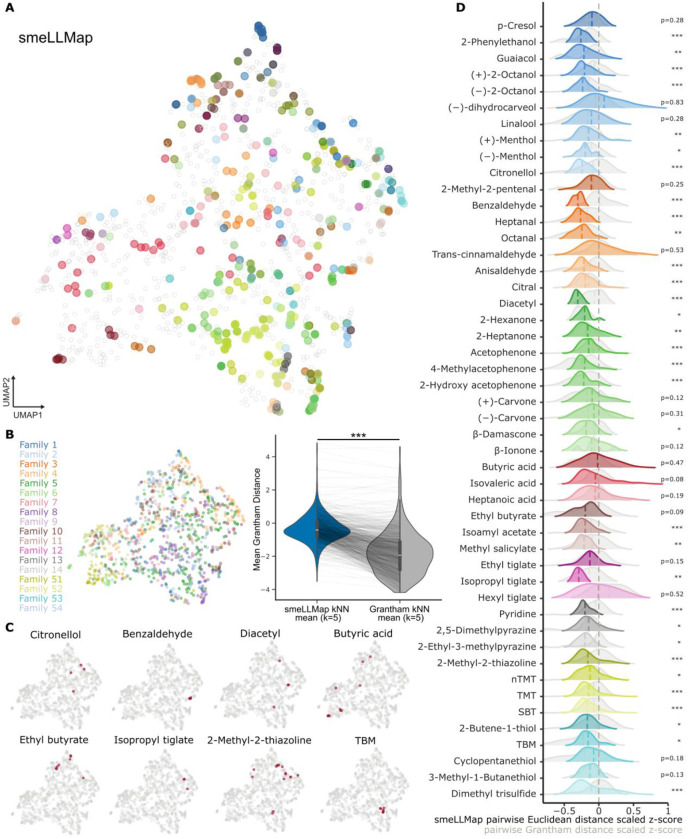
SmeLLMap captures odor-specific relationships among odorant receptors. **A**. Left: smeLLMap visualization of OR embeddings using UMAP dimensionality reduction. Each point represents a single OR; receptors with no detected responses are shown in hollow grey, while responding receptors are overlayed by odorant colors to which they respond based on pS6-IP activation labels. Right: individual smeLLMap subplots highlighting ORs response to a single odorant. **B**. Left: smeLLMap representation of ORs by family. Right: Violin plots show Grantham distance between neighbor distributions across receptors in smeLLMap. line indicates matched random pairs. Paired t-test, *p* = 1.05 × 10^−35^. **C**. smeLLMap representation of individual ORs activated (labeled in red) by specific odorant clusters. **D**. Violin plots of pairwise Euclidean distances between CNN-derived embeddings of ORs responding to the same odorant. Statistical significance was assessed using permutation tests with randomly sampled OR groups matched for group size per odorants. Grey violins denote the corresponding pairwise Grantham distance distributions for the same OR sets.

We first examined the global organization of the learned representation using UMAP dimensionality reduction for visualization. The resulting two-dimensional embedding, which we term smeLLMap, provides a spatial map of the OR landscape in which each point corresponds to a single receptor and its position reflects similarity in the learned odor-response representation, with colors indicating ligand selectivity (Fig. 4A and fig S5). At a global level, receptors do not strongly segregate by canonical family assignments (Fig. 4B). Consistent with this, functional organization emerges as the dominant structure. When receptors responding to individual odorants are visualized, they form distinct and coherent clusters within smeLLMap (Fig. 4C and fig. S5), indicating that the learned representation groups ORs according to shared ligand selectivity rather than phylogenetic relatedness. To quantify the relationship between embedding proximity and sequence similarity, we computed Grantham distance between 5 k nearest neighbors (kNN) derived from smeLLMap in comparison with those defined by amino acid-based neighbors for each ORs. Nearest neighbors in the smeLLMap exhibited significantly higher Grantham distances than amino acid derived neighbors (Fig. 4B). This result demonstrates that local organization in the smeLLMap is not solely driven by sequence similarity, but instead reflects alternative features captured by the model.

We next asked whether model embedding-based similarity better reflects shared ligand selectivity than primary amino acid sequence similarity. To address this, we computed pairwise Grantham distances between ORs responding to the same odor and compared these distributions with Euclidean distances measured in the smeLLMap. Subsequently, for 41 of 48 odorants, responding ORs show higher similarity between learned embedding than amino acid similarity (Fig. 4D and table S2). Together, these patterns and difference indicates that the learned embedding representation captures functional similarities between receptors that are not apparent from primary amino acid sequence similarity alone, highlighting the added value of the CNN-derived structural embedding over sequence-based metrics.

### Chemical and Perception Inference

**Figure 5.**
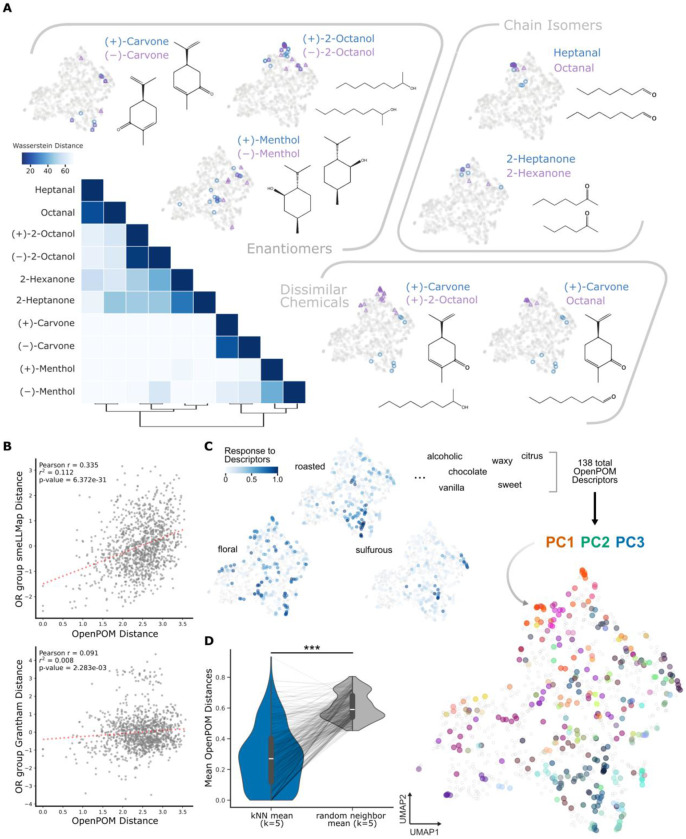
SmeLLMap captures chemical and perceptual similarity relationships among odorant receptors. **A**. smeLLMap reflects chemical relationships between odorants. Left: Heatmap of Wasserstein distances between distributions of OR embeddings for each odorant, clustered by similarity. Right: Representative smeLLMap projections illustrating receptor distributions for selected odorant pairs, including enantiomers, structurally related compounds (heptanal vs. octanal; 2-heptanone vs. 2-hexanone), and chemically dissimilar odorants ((+)-carvone vs. octanal). **B**. Quantitative comparison between receptor embedding derived distances and perception-informed chemical space. Top: Pearson correlation between pairwise Wasserstein distances of responding OR groups in smeLLMap and corresponding Euclidean distances in OpenPOM space across all 48 odorants. Bottom: Equivalent analysis using Grantham sequence distances in place of embedding distances, showing reduced correspondence with OpenPOM-derived relationships. **C**. Mapping perceptual features onto receptor embedding space. Left: Selected OpenPOM descriptors (e.g., floral, roasted, sulfurous) projected onto smeLLMap, revealing spatially localized receptor tuning. Right: Integration of all 138 OpenPOM descriptors, reduced by principal component analysis and visualized as RGB color channels (PC1-PC3). **D**. Local neighborhood structure in embedding space reflects perceptual similarity. For each receptor, the mean OpenPOM Euclidean distance of its five nearest neighbors in smeLLMap is compared to a random expectation. Violin plots show distributions across receptors; line indicates matched random pairs. Paired t-test, *p* = 6.46 × 10^−96^.

We next asked whether the smeLLMap reflects relationships between odorants, despite the model having no explicit access to ligand chemical features during training. To address this, we first examined whether receptors responding to chemically similar odorants occupy similar regions in the learned latent space. For each odorant, the set of responding OR embeddings was treated as a distribution, and pairwise distances between odorants were quantified using Wasserstein distance (Fig. 5A and fig. S6). Odorants with high structural similarity exhibited significantly smaller distances between their corresponding receptor distributions. This was observed for closely related compounds such as stereoisomeric pairs, as well as homologous series including heptanal and octanal, which differ by a single carbon unit (Fig. 5A and fig. S6). In contrast, chemically dissimilar odorants, such as octanal and (+)-carvone, showed substantially larger distances between their respective receptor distributions. These results indicate that smeLLMap can infer chemical similarity through receptor organization, even in the absence of explicit ligand descriptors.

While these relationships highlight the ability of the model to recover chemical similarities between enantiomer and chain isomer odorants, we next asked whether this organization extends beyond chemical features to reflect perceptual similarity. To test this, for each pair of odorants we computed the distance between their responding OR distributions in smeLLMap and compared these values to the corresponding distances derived from OpenPOM (27, 45) space. Across all 48 odorants, we observed a significant positive correlation between receptor embedding distance and perceptual distance (*r*^2^=0.112, *p*=6.372e-31) (Fig. 5B and fig. S6A), indicating that odorants perceived as more similar by humans panels tend to be represented by similar ORs represented with regions of receptor space. Notably, this relationship was not clear when using amino acid sequence-based Grantham distances between responding ORs (*r*^2^=0.007, *p*=2.283e-3) (Fig. 5B and fig. S6B), demonstrating that primary sequence similarity alone does not capture perceptual organization.

To further visualize how perceptual features are represented across receptor space, we projected individual OpenPOM-derived odor descriptors onto smeLLMap. By mapping specific odor descriptors, such as floral, roasted and sulfurous qualities, revealed localized clusters of receptors preferentially associated with descriptors (Fig. 5C). We next examined whether this organization could be observed at a global level by integrating the full set of 138 OpenPOM descriptors. Utilizing dimensionality reduction of these perceptual features followed by projection onto smeLLMap, with each principal component axis as a representation RGB color space revealed further clustering of colored gradients. Likely indicating that perceptual features may also be organized and inferred by the OR space, highlights a structured encoding in which different regions of receptor space are tuned to distinct combinations of perceptual features. To quantitatively assess whether local neighborhoods in smeLLMap reflect shared perceptual tuning, we computed kNN relationships and compared the OpenPOM (27, 45) representations of neighboring receptors. Because our analyses relate mouse receptor activation to human perception derived features, they should be interpreted with appropriate caution. Nevertheless, receptors exhibited significantly greater similarity in perceptual representation to their nearest neighbors than random (Fig. 5D), supporting the idea that smeLLMap captures perceptually relevant organization.

Together, these results demonstrate that smeLLMap captures not only chemical similarity but also perceptual relationships between odorants. This organization reflects encoding in which receptor structure gives rise to functional groupings that align with both molecular features and human odor perception. This finding suggests that perceptual similarity may emerge, at least in part, from the structure of receptor activation patterns encoded within the OR binding cavity.

### Feature Importance

**Figure 6.**
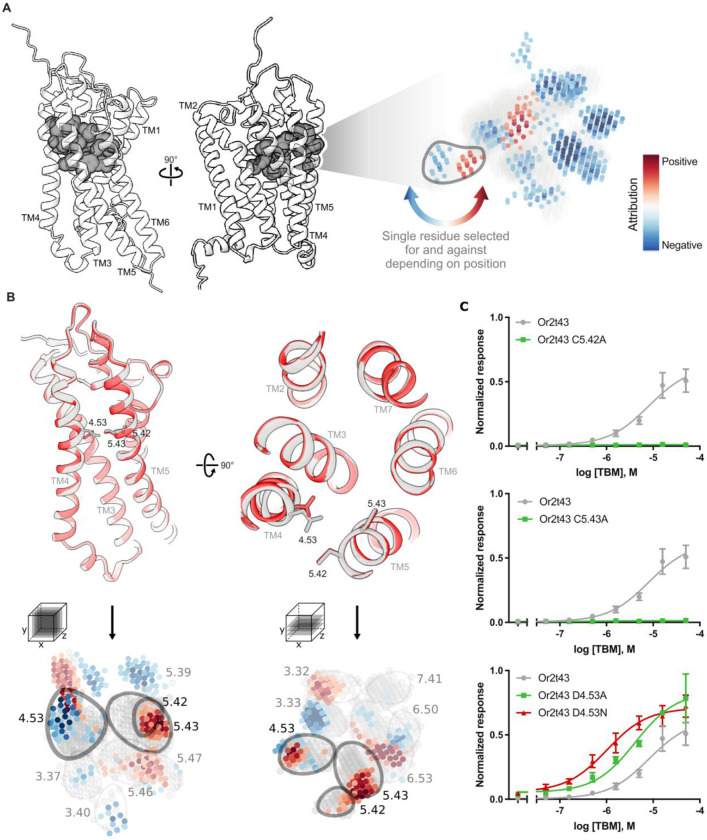
Voxel-level attribution identifies structural determinants of odor selectivity. **A**. Structural and voxel-based representations of olfactory receptor binding cavities. Left, occupancy representation of amino acid residues defining the extracted canonical binding cavity mapped onto the OR structure, shown from two viewing angles. Right, corresponding three-dimensional voxel saliency map, highlighting spatial regions within the cavity that contribute most strongly to model predictions. **B**. Top: Predicted AlphaFold3 structures of native Or2t43 and Or2t43D4.43N mutant with side and top view orientation. Bottom: Stacks aggregated saliency maps for receptors responding to tert-butyl mercaptan (TBM). Left and middle, orthogonal slice views through the averaged voxel attribution map reveal conserved high-importance regions within the binding cavity. **C**. functional validation by mutagenesis of residues D4.53, C5.42, and C5.43 in Or2t43, assessed using a real-time cAMP luciferase assay. Data are shown as mean ± s.d. from *n* = 3 independent experiments.

While the smeLLMap captures the global organization of receptor-odorant relationships, we next sought to identify which specific structural features most strongly influenced individual model predictions. To this end, we applied Integrated Gradients (IG), which attributes a model’s output to input voxels by measuring how the prediction changes as structural information is gradually introduced from a baseline representing the absence of receptor structure. Voxels with large attribution magnitudes, either positive or negative, exert the strongest influence on the prediction, yielding a three-dimensional importance map over the binding cavity that highlights features that promote or disfavor ligand binding (Fig. 6A). Importantly, each voxel in our representation encodes both spatial location and residue-level biochemical features. As a result, voxel attribution reflects the joint contribution of three-dimensional positioning and physicochemical identity, rather than isolating either factor in abstraction. This integrated representation is advantageous, as ligand recognition in ORs depends on the precise spatial arrangement of chemically specific residues within the binding pocket. Attribution scores therefore capture how geometry and chemistry act together to shape receptor selectivity (Fig. 6A).

Notably, when attributions were aggregated across multiple receptors responding to the same odorant, importance did not simply track residue identity. Instead, the structural conformation of those residues appeared to influence their contribution to ligand binding. The same residue position could contribute positively or negatively depending on whether its side chain projected inward to constrict the cavity or outward to expand it, likely excluding or permitting ligand access (Fig. 6A). Together, these results indicate that odorant selectivity is governed not only by which residues are present, but by how their three-dimensional positioning modulates cavity accessibility.

We next asked whether this combined embedding and voxel-level attribution framework could be used to guide odorant-receptor activation. As a case study, we focused on tert-butyl mercaptan (TBM), a sulfur-containing odorant widely used as a natural gas additive due to its strong detectability.

Guided by the IG attributions, we engineered targeted mutations in Or2t43 and assessed their responses to TBM (Fig. 6B). Although Or2t43 is robustly activated by TBM in its native form, mutation of either C5.42 or C5.43 resulted in a complete loss of activation, consistent with disruption of the predicted sulfur-binding coordination site. In contrast, substitution of D4.53 with alanine, reducing side-chain size and polarity, or with asparagine, maintaining steric occupancy while altering charge, both increased TBM responsiveness to TBM. These reciprocal effects are consistent with the directionality of the model’s attribution scores and indicate that the highlighted residues exert functionally meaningful influence on ligand sensitivity. In addition, the model identified residue D4.53 as negatively associated with TBM responsiveness, suggesting a potential inhibitory role at this position.

To further examine the structural basis of these effects, we generated AlphaFold3 models of the corresponding Or2t43 mutants. Comparison of the predicted structures revealed that mutation of D4.53 to asparagine results in a repositioning of the side chain within the binding cavity. Notably, the mutated N4.53 side chain extends into a spatial region immediately adjacent to, but distinct from, the original asparagine position, consistent with the observed increase in TBM responsiveness. These structural changes align with the attribution-derived importance map, providing support that local rearrangements within the binding cavity can modulate ligand sensitivity in a predictable manner.

Together, these results demonstrate that our interpretable Spatial LLM framework not only captures the global organization of odor-receptor relationships but also provides mechanistic insight at residue-level resolution. By linking structural context, learned embeddings, and voxel-level attribution, the model enables rational prediction of functionally relevant mutations and supports targeted modulation of receptor sensitivity to specific odorants.

## Discussion

This study demonstrates that OR-ligand selectivity can be learned and predicted through a structure-centered framework that explicitly models the three-dimensional organization and sequence-informed composition of the receptor binding cavity. By integrating in vivo pS6-IP-Seq activation measurements with spatial learning, we show that the canonical binding cavity contains sufficient information to organize receptor responses across chemically diverse odorants (Fig. 3B,D and 4D). These findings indicate that the determinants of ligand selectivity are encoded within the binding pocket itself, even without explicit modeling of alternative or allosteric interaction sites, thereby addressing a long-standing challenge in linking receptor variation to interpretable chemical recognition.

A key conceptual advance of this work is the demonstration that functional similarity among ORs is better captured by learned structural embeddings than by primary sequence similarity alone (Fig. 4B). While traditional sequence-based metrics and protein language model embeddings reflect evolutionary relationships, they fail to fully explain why distantly related receptors can converge on similar ligand selectivity. In contrast, the CNN-derived embedding organizes receptors according to shared response profiles, revealing functional neighborhoods that spans beyond phylogenetic relationship. This result suggests that ligand selectivity is governed by higher-order structural constraints, such as cavity geometry and residue positioning, that are not apparent from sequence comparisons.

Notably, this organization extends beyond chemical similarity to reflect perceptual relationships between odorants (Fig. 5A–D and fig. S6). Despite being trained without explicit ligand features, the learned embedding correlates with perceptual distances derived from OpenPOM (45), a model trained on chemical molecules to predict odor perception, whereas amino acid sequence-based distances do not (Fig. 5B). This correspondence suggests that receptor binding cavity encode features that can be used to infer odor perceptual similarity. At the same time, these comparisons should be interpreted with appropriate caution. The receptor structures and activation data in this study are derived from mouse, whereas OpenPOM reflects human perceptual judgments. Rather than implying a direct mapping between mouse receptor activity and human perception, this result points to shared, cross-species constraints in how chemical features are transformed into receptor-level representations. Such constraints may arise from conserved structural principles governing receptor-ligand interactions. Future work integrating human OR repertoires, alongside matched perceptual datasets, will be important to determine the extent to which receptor-level features directly encode perceptual dimensions. Establishing this link will help clarify whether the observed alignment reflects a generalizable organizing principle or emerges from partial overlap between species-specific receptor spaces.

Importantly, the interpretability of this representation allows functional predictions to be traced back to specific structural features (Fig. 6A–C). Integrated Gradients attribution revealed that both positively and negatively contributing voxels shape ligand responsiveness, highlighting residues that promote binding as well as those that disfavor activation. The observation that identical residues can exert opposite effects depending on their three-dimensional orientation underscores that odorant recognition is fundamentally a spatial phenomenon (Fig. 6A). These results support a model in which receptor tuning arises from the precise arrangement of residues within the binding cavity, rather than from residue identity alone.

Given the dynamic nature of OR activation, the ability of static AlphaFold3 predicted structures to support prediction and attribution is notable. While receptor conformational and ligand-induced rearrangements undoubtedly contribute to signaling, our results indicate that determinants of selectivity are already encoded in the active-state architecture of the binding pocket structure and ESM features (Fig. 3E). Although this framework does not explicitly model OR secondary or allosteric binding sites, the predictive performance suggests that such interactions, when present, either act through modulation of the canonical cavity or play a secondary role in determining primary ligand selectivity. Alternatively, the current framework may also suggest the frequency of binding outside of canonical binding cavity occur in relatively limited subset of receptors such that the overall statistical framework remains significant. Additionally, by encoding amino acid features with ESM embeddings may also enables the feature to capture long-range dependencies that could be outside of the selected binding cavity itself (fig. S2). Future incorporation of receptor dynamics, alternative conformational states, or ligand-induced structural shifts may further refine predictions, but may not be required to recover core selectivity principles.

Beyond mechanistic insight, this study provides a scalable strategy for interrogating OR function at a systems level. The use of pS6-IP-Seq, coupled with an activation metric designed to minimize transcript abundance and variance biases, enabled broad and relatively unbiased sampling of receptor-odorant interactions in vivo (Fig. 1A–F). The resulting dataset supports a model that generalizes across receptors and odorants, including cases with sparse activation data (Fig. 3D), highlighting the importance of carefully designed functional metrics in enabling downstream computational modeling.

While the present study focuses on single odorants at fixed concentrations, several extensions could substantially broaden its scope. Expanding chemical coverage would improve representation of sparsely sampled ligand classes, while incorporating concentration dependence could reveal how cavity features govern sensitivity and dynamic range. Natural olfactory stimuli are typically complex mixtures (4, 19), and extending the framework to model mixture responses or competitive interactions between ligands represents an important next step. Although caution is warranted when interpreting pS6-IP-Seq data, as odorants used for stimulation may be metabolized within the olfactory mucus before binding to ORs (51), integrating explicit ligand representations alongside receptor structures may further enable dissection of receptor-ligand complementarity and cross-reactivity.

The implications of this work extend beyond olfaction. ORs belong to the GPCR superfamily, which encompasses a wide range of receptors involved in sensory perception, neurotransmission, and pharmacological signaling. Demonstrating that structural features alone can organize receptor function in a highly diverse GPCR family suggests that similar approaches may be applicable to other receptors lacking extensive experimental characterization. In this context, the framework introduced here provides a generalizable strategy for linking receptor structure to ligand selectivity in an interpretable and predictive manner.

In summary, the central of this study is that OR function is encoded in the three-dimensional structure of the binding cavity in a way that can be learned, interpreted, and experimentally validated. By incorporating sequence-centric descriptions to structure-informed representations, this work contributes toward a predictive, mechanistic understanding of chemical sensing. As structural models, functional data, and computational methods continue to converge, this approach lays the groundwork for quantitative and ultimately generative models of olfactory coding.

## Materials and Methods

### Phosphorylated S6 ribosomal immunoprecipitation sequencing (pS6-IP-Seq)

Mice used for pS6-IP were ~3 weeks old, mixed sex, and littermates. Mice were killed by CO_2_ asphyxiation and cervical dislocation. Olfactory tissue was rapidly dissected in Buffer B (2.5 mM HEPES KOH pH 7.4, 0.63% glucose, 100 μg/mL cycloheximide, 5 mM sodium fluoride, 1 mM sodium orthovanadate, 1 mM sodium pyrophosphate, 1 mM β-glycerophosphate, in Hank’s balanced salt solution). Tissue pieces were then minced in 1.35 mL Buffer C (150 mM KCl, 5 mM MgCl_2_, 10 mM HEPES KOH pH 7.4, 0.100 μM Calyculin A, 2 mM DTT, 100 U/mL RNAsin, 100 μg/mL cycloheximide, protease inhibitor cocktail, 5 mM sodium fluoride, 1 mM sodium orthovanadate, 1 mM sodium pyrophosphate, 1 mM β-glycerophosphate)and subsequently transferred to homogenization tubes for steady homogenization at 250 rpm three times and at 750 rpm nine times at 4 °C. Samples were then transferred to a 1.5 mL LoBind tube (Eppendorf 022431021) and clarified at 2000xg for 10 min at 4 °C. The low-speed supernatant was transferred to a new tube on ice, and 90 μL of NP40 (Sigma 11332473001) and 90 μL of 1,2-diheptanoyl-sn-glycero-3-phosphocholine (DHPC, Avanti Polar Lipids 850306P, 100 mg/0.69 mL) were added to this solution. This solution was mixed and then clarified at a max speed (17,000xg) for 10 min at 4 °C. The resulting high-speed supernatant was transferred to a new tube where 20 μL was saved and transferred to a tube containing 350 μL buffer RLT. To the remainder of the sample, 1.3 μL of 100 μg/mL cycloheximide, 27 μL of phosphatase inhibitor cocktail (250 mM sodium fluoride, 50 mM sodium orthovanadate, 50 mM sodium pyrophosphate, 50 mM β-glycerophosphate) and 6 μL of anti-pS6 antibody (Cell Signaling D68F8) were added. The sample was gently rotated for 90 min at 4 °C. To prepare beads, 100 μL of beads (Invitrogen 10002D) was washed three times with 900 μL of buffer A (150 mM KCl, 5 mM MgCl_2_, 10 mM HEPES KOH pH 7.4, 10% NP40, 10% BSA), and once with 500 μL of buffer C. Sample homogenate was added to the beads and incubated with gentle rotation for 60 min at 4 °C. Following incubation, beads were washed with four times with 700 μL of buffer D (350 mM KCl, 5 mM MgCl_2_, 10 mM HEPES KOH pH 7.4, 10% NP40, 2 mM DTT, 100 U/mL RNAsin, 100 μg/mL cycloheximide, 5 mM sodium fluoride, 1 mM sodium orthovanadate, 1 mM sodium pyrophosphate, 1 mM β-glycerophosphate). During the final wash, beads were moved to room temperature, wash buffer was removed, and 350 mL of buffer RLT was added. Beads were incubated in buffer RLT for 5 min at room temperature. Buffer RLT containing immunoprecipitated RNA was then eluted and stored at −80 °C until clean up using a kit (Qiagen 74004). cDNA was generated using 11 rounds of amplification with 10 ng RNA input. DNA libraries were prepared using a half-sized Nexterra XT DNA Library Preparation Kit (Illumina 15032354) protocol as per the manufacturer’s guidelines. Libraries were sequenced on either HiSeq 2000/2500 (50 base pair single read mode) or NextSeq 500 (75 base pair single read mode) with 6–12 pooled indexed libraries per lane.

### RNA-Seq alignment, quantification, and differential expression analysis

Reads were aligned against a modified GRCm38.p6 (M25) reference, in which we deleted ENSMUSG00000116179 (Olfr290), using STAR (52) with --outFilterMultimapNmax 10. Reads mapping to Olfr290 were inferred from ENSMUSG00000070459, with the rationale that this gene model included ENSMUSG00000116179 plus untranslated regions. Gene-level read quantification was done using RSEM (53). Differential expression analysis was performed against all genes using EdgeR (54). Gene nomenclature was retrieved from BioMart (55). Intact *Olfr* genes with identifiable sequences were filtered, and p-values were then re-corrected by FDR. Only ORs exhibiting odor response to at least one of the tested odorants (log_2_FC > 0 and FDR < 0.05) were considered. A total of 555 ORs responded across the 72 different odorants at various concentrations. A total of 375 ORs were responsive to unique odorants at the lowest tested concentrations. Raw and processed RNA-Seq datasets generated as part of this study are available from NCBI GEO at accession GSE185415.

### Source of odorants

The following odors and concentrations were used for molecular response profiling and model training: 1% p-Cresol (Sigma C85751), 1% 2-phenylethanol (Sigma 77861), 1% guaiacol (Sigma G10903), 10% (+)-2-octanol (Sigma O4504), 10% (−)-2-octanol (Sigma 147990), 1% linalool (Sigma L2602), 1 M (+)-menthol (Sigma 224464), 1 M (−)-menthol (Sigma M2780), 1% citronellol (Sigma W230915), 1% 2-methyl-2-pentenal (Sigma 294667), 1% benzaldehyde (Sigma W212717), 1% heptanal (Sigma W254002), 1% octanal (Sigma O5608), 1% trans-cinnamaldehyde (Sigma C80687), 0.01% anisaldehyde (Sigma A88107), 0.01% citral (Sigma W230316), 1% ethyl butyrate (Sigma W242713), 1% isoamyl acetate (Sigma 306967), 1% methyl salicylate (Sigma W274502), 1% diacetyl (Sigma W237027), 1% 2-hexanone (Sigma 103004), 1% 2-heptanone (Sigma 537683 (41)), 0.01% acetophenone (Sigma W200910), 1% 4-methylacetophenone (Sigma W267708), 1% (+)-carvone (Sigma 22070), 1% (−)-carvone (Sigma 22060), 1% β-damascone (Sigma W324300), 1% β-ionone (Sigma W259525), 1% pyridine (Sigma 270970), 1% 2,5-dimethylpyrazine (Sigma 175420 (41)), 1% 2-ethyl-3-methylpyrazine (Sigma W315508), 0.01% 2-butene-1-thiol (CheMall Corp OR116574), 1% tert-butyl mercaptan (2-methyl-2-propanethiol; Sigma 109207), 0.01% cyclopentanethiol (Sigma W326208), 1% 3-methyl-1-butanethiol (Sigma W385808), 1% dimethyl trisulfide (Sigma W327506), 1% 2-methyl-2-thiazoline (Sigma M83406), 1% 2,4,5-trimethylthiazole (nTMT; Sigma 219185), 0.01% 2,4,5-trimethyl-4,5-dihydrothiazole (TMT, synthesized (56)), 0.01% 2-sec-butyl-4,5-dihydrothiazole (SBT; synthesized (41)), 1% ethyl tiglate (Sigma W246000), 1% isopropyl tiglate (Sigma W322903), and 1% hexyl tiglate (Sigma W500909).

### Chemical space estimation

To estimate chemical space, we first identified 4680 small molecules commonly found in foods and fragrances from http://www.thegoodscentscompany.com/ (43). Smiles strings for these molecules and the 48 in the test odor set were then downloaded from PubChem, and Morgan-algorithm based fingerprints were calculated using Rdkit (44, 57). Chemical space was estimated by PCA dimensionality reduction on all molecules.

To complement this structure-based representation with a perceptual framework, we additionally embedded all molecules using OpenPOM(27, 45), a message-passing neural network trained to predict human odor perception from chemical structure. The resulting OpenPOM feature vectors provide a high-dimensional representation of perceptual odor space derived from human panel data (58). These embeddings were used to assess perceptual diversity of the odor panel, perform pairwise distance comparisons, and relate receptor embedding organization to perceptual similarity in downstream analyses.

### Receptor alignment and space estimation

Mouse ORs were aligned to one another using the MAFFT E-INS-I method with manual refinements (59). The resulting alignment file was subjected to ModelTest-NG to identify ideal amino acid substitution models (60). Receptor pairwise similarity was calculated by summing amino acid differences at each position by Grantham’s amino acid distances (61). Multidimensional scaling on all ORs.

### Structural Model Generation

All mouse olfactory receptor (OR) protein sequences were compiled together with mini-Gα peptide definitions into standardized JSON configuration files. For each receptor, five independent structural predictions of the OR–miniGα complex were generated using AlphaFold-Multimer (13), capturing variability in receptor conformation under identical modeling conditions. In parallel, five ORs with respective ligands with available cryo-EM structures (OR51E2, consOR1, consOR2, consOR4, consOR51) were processed through the same workflow to serve as experimentally grounded structural references. Following structural prediction, all OR complexes were placed into a common coordinate frame using TM-align (62), ensuring consistent spatial orientation of transmembrane helices and intracellular domains across receptors. Internal solvent-accessible pockets were then identified using pyKVFinder (49), which outputs cavity coordinates. This procedure was applied uniformly to predicted models, cryo-EM structures, and later to ligand-bound complexes.

### Canonical Binding Cavity Identification

To determine the biologically relevant ligand binding region, we incorporated receptor–odorant activation data derived from pS6-IP assays. For each experimentally activated receptor–ligand pair, AlphaFold3 was used to model receptor–miniGα–ligand complexes, followed by cavity detection with pyKVFinder. Ligand-occupied pockets were spatially compared across cryo-EM receptors, and regions demonstrating ≥50% overlap across ligand-bound cavities were defined as the canonical binding cavity. This region represents the reproducibly utilized ligand-engagement space across structurally diverse ORs.

The canonical binding cavity mask was then projected back onto ligand-free OR models for the entire repertoire. For each receptor, pyKVFinder-defined cavities were filtered relative to the canonical binding cavity; regions with <80% spatial overlap were discarded. This filtering process preserved a single, standardized binding cavity per receptor while removing allosteric, non-functional or receptor-specific peripheral pockets. Individual receptor’s cavity surrounding amino acid then defined by less than 5 Å. The resulting structures consist of coordinates of each OR binding cavity and amino acid coordinates.

### Voxelization and Feature Encoding

For each receptor, all residues spatially lining the canonical binding cavity were extracted to construct a localized structural representation. The cavity volume of each receptor was then discretized into a 32 × 32 × 32 voxel grid, maintaining 1Å spatial resolution, such that each voxel encodes a fixed physical region of three-dimensional space surrounding the binding pocket. For every voxel containing protein atoms, the residue occupying that spatial position was assigned as the voxel occupant. To provide a rich biochemical and evolutionary descriptor beyond atomic coordinates, the reduced ESM sequence embedding vector corresponding to that residue was inserted as the voxel feature representation. Voxels not occupied by residues were assigned a null background vector. This procedure resulted in a structured, spatially aligned, and biologically informed voxel map for each receptor. In this final representation, every OR is encoded as a voxelized binding cavity populated with residue-level embedding features, providing a standardized structural input for downstream convolutional neural network training and odor selectivity modeling.

### Heterologous luciferase assay

Hana3A cells; which stably express Golf, RTP1, RTP2, and REEP1; were grown in minimum essential medium eagle (MEM; Corning 10-010-CV) containing 10% Fetal Bovine Serum (FBS; vol/vol; Gibco 16000-044), penicillin-streptomycin (Sigma-Aldrich P4333), and amphotericin B (Gibco 15290018). Cells were cultured and incubated at 37°C, 5% CO_2_, and saturated humidity for use with the Dual-Glo Luciferase Assay (Promega E2980)(63, 64). Cells were plated at 20-25% confluence on poly-D-lysine-coated 96-well plates (Corning 3843) overnight. After overnight incubation, cells were transfected with 6 mL of MEM containing 10% FBS, 0.5 μg SV40-RL (Promega E2980), 1 μg CRE-Luc (Promega E2980), 0.5 μg mouse RTP1s, 0.25 μg M3 muscarinic receptor, 0.5 μg of Rho-tagged receptor plasmid DNA, and 20 μg Lipofectamine 2000 (Invitrogen 11668019) per plate. Transfection medium was divided equally among the wells so that each OR-odorant combination could be conducted in triplicates. The following day, cells were incubated with 25μL of odorant solution diluted in CD-293 (Gibco 11913-019) containing 30 μM CuCl_2_ (Sigma-Aldrich C-6641) and 2 mM glutamine (Gibco 25030-081) for 3.5 hours. cAMP-driven firefly Luciferase luminescence (Luc) was used to assess OR activation, and SV40-driven *Renilla* Luciferase luminescence (Ren) was used to control for variation in cell viability within wells. Cell luminescence was read by a POLARstar OPTIMA (BMG Labtech) luminometer, and normalized response values were calculated using the formula (Luc-400)/(Ren-400). ORs were considered responsive *in vitro* if ANOVA *p*-value was < 0.05 and ANOVA with post-hoc Dunnet’s test correction *p*-adjusted was < 0.05 for at least 2 of the tested odor concentrations using the R package DescTools (v0.99.42). Log-logistic 4-parameter dose response curves were fit to the data using the R package drc (v3.0-1). *In vitro* responses were compared to *in vivo* responses by subtracting mean ligand-independent activity (luciferase values of ORs with no odor stimulation) from each of the ligand stimulated data points and summing. Scaled summed (+)-enantiomer responses were divided by scaled summed (−)-enantiomer responses and log_2_ transformed for comparison to log_2_FC (+)/(−) *in vivo* enrichments.

### Data analysis

Analysis were performed in Python 3.8. Custom scripts for data analysis and visualization built using open-source python libraries (pandas, numpy, matplotlib, plotly, sklearn, scipy, seaborn and itertools). Scripts to replicate data analysis are available on github (https://github.com/Justice-Lu/OR_learning).

### Statistical analysis

Experimental data are presented as mean ± standard deviation (SD), based on results from at least three independent experiments. Statistical analysis was conducted in Python 3.11.13 using SciPy v1.16.0. Paired comparisons (e.g., samples with different condition measured in the same experiment) were analyzed using the paired Student’s t-test (ttest_rel). For unpaired comparisons between two independent groups, the non-parametric Wilcoxon rank-sum test (ranksums) was applied. A table with all the statistical comparison values and description can be found at Table 3. A two-tailed p-value less than 0.05 was considered statistically significant. Significance levels are denoted as follows: p < 0.05 (*), p < 0.01 (**), p < 0.001 (***), and p ≥ 0.05 (n.s., not significant).

## Supplementary Material

1

This is a list of supplementary files associated with this preprint. Click to download.

• Naturesupplementarymaterials.docx

## Data Availability

All code used for data processing, structural modeling, and machine learning is publicly available at GitHub: https://github.com/Justice-Lu/OR_learning/tree/master. Raw and processed RNA-sequencing data generated in this study have been deposited in the NCBI Gene Expression Omnibus (GEO) under accession number GSE185415. Additional data supporting the findings of this study are available from the corresponding author upon reasonable request.

## References

[1] Bushdid C., Magnasco M. O., Vosshall L. B., Keller A., Humans Can Discriminate More than 1 Trillion Olfactory Stimuli. Science 343, 1370–1372 (2014). 24653035 10.1126/science.1249168 PMC4483192

[2] Buck L., Axel R., A novel multigene family may encode odorant receptors: a molecular basis for odor recognition. Cell 65, 175–187 (1991). 1840504 10.1016/0092-8674(91)90418-x

[3] Niimura Y., Matsui A., Touhara K., Extreme expansion of the olfactory receptor gene repertoire in African elephants and evolutionary dynamics of orthologous gene groups in 13 placental mammals. Genome Res 24, 1485–1496 (2014). 25053675 10.1101/gr.169532.113 PMC4158756

[4] Tsukahara T. A transcriptional rheostat couples past activity to future sensory responses. Cell 184, 6326–6343.e6332 (2021). 34879231 10.1016/j.cell.2021.11.022 PMC8758202

[5] Billesbølle C. B. Structural basis of odorant recognition by a human odorant receptor. Nature 615, 742–749 (2023). 36922591 10.1038/s41586-023-05798-y PMC10580732

[6] de March C. A. Engineered odorant receptors illuminate the basis of odour discrimination. Nature 635, 499–508 (2024). 39478229 10.1038/s41586-024-08126-0 PMC13504986

[7] Choi C. Understanding the molecular mechanisms of odorant binding and activation of the human OR52 family. Nat Commun 14, 8105 (2023). 38062020 10.1038/s41467-023-43983-9 PMC10703812

[8] Han X. Mechanistic insights into fatty acid odor detection mediated by class II olfactory receptors. Cell, (2026). 10.1016/j.cell.2025.12.018 41570821

[9] Ge X.-Y. Identification of Or5v1/Olfr110 as an oxylipin receptor and anti-obesity target. Cell, (2026). 10.1016/j.cell.2025.12.016 41570820

[10] Wang T. Structural decoding of reversible covalent linkage of odorants in human olfactory receptor OR6A2. Cell, (2026). 10.1016/j.cell.2025.12.017 41570822

[11] Gil M. Structural basis of odorant recognition by a mammalian class II odorant receptor. Sci Adv 12, eaeb9026 (2026). 41894497 10.1126/sciadv.aeb9026 PMC13025043

[12] Jumper J. Highly accurate protein structure prediction with AlphaFold. Nature 596, 583–589 (2021). 34265844 10.1038/s41586-021-03819-2 PMC8371605

[13] Abramson J. Accurate structure prediction of biomolecular interactions with AlphaFold 3. Nature 630, 493–500 (2024). 38718835 10.1038/s41586-024-07487-w PMC11168924

[14] Lin Z. Evolutionary-scale prediction of atomic-level protein structure with a language model. Science 379, 1123–1130 (2023). 36927031 10.1126/science.ade2574

[15] Wald G., The Receptors of Human Color Vision: Action spectra of three visual pigments in human cones account for normal color vision and color-blindness. Science 145, 1007–1016 (1964). 14172613 10.1126/science.145.3636.1007

[16] Schnapf J., Kraft T., Baylor D., Spectral sensitivity of human cone photoreceptors. Nature 325, 439–441 (1987). 3808045 10.1038/325439a0

[17] Buck L., Axel R., A novel multigene family may encode odorant receptors: a molecular basis for odor recognition. Cell 65, 175–187 (1991). 1840504 10.1016/0092-8674(91)90418-x

[18] Bozza T. Mapping of class I and class II odorant receptors to glomerular domains by two distinct types of olfactory sensory neurons in the mouse. Neuron 61, 220–233 (2009). 19186165 10.1016/j.neuron.2008.11.010 PMC3013286

[19] Horgue L. F. Transcriptional adaptation of olfactory sensory neurons to GPCR identity and activity. Nature Communications 13, 2929 (2022). 10.1038/s41467-022-30511-4 PMC9132991 35614043

[20] del Mármol J., Yedlin M. A., Ruta V., The structural basis of odorant recognition in insect olfactory receptors. Nature 597, 126–131 (2021). 34349260 10.1038/s41586-021-03794-8 PMC8410599

[21] Baek M. Accurate prediction of protein structures and interactions using a three-track neural network. Science 373, 871–876 (2021). 34282049 10.1126/science.abj8754 PMC7612213

[22] Lin Z. Language models of protein sequences at the scale of evolution enable accurate structure prediction. BioRxiv 2022, 500902 (2022).

[23] Lötsch J., Kringel D., Hummel T., Machine Learning in Human Olfactory Research. Chem Senses 44, 11–22 (2019). 30371751 10.1093/chemse/bjy067 PMC6295796

[24] Mayhew E. J. Transport features predict if a molecule is odorous. Proc Natl Acad Sci U S A 119, e2116576119 (2022). 35377807 10.1073/pnas.2116576119 PMC9169660

[25] Khan R. M. Predicting odor pleasantness from odorant structure: pleasantness as a reflection of the physical world. J Neurosci 27, 10015–10023 (2007). 17855616 10.1523/JNEUROSCI.1158-07.2007 PMC6672642

[26] Nozaki Y., Nakamoto T., Predictive modeling for odor character of a chemical using machine learning combined with natural language processing. PLoS One 13, e0198475 (2018). 29902194 10.1371/journal.pone.0198475 PMC6002022

[27] Lee B. K. A principal odor map unifies diverse tasks in olfactory perception. Science 381, 999–1006 (2023). 37651511 10.1126/science.ade4401 PMC11898014

[28] Shuvaev S., Tran K., Samoilova K., Mascart C., Koulakov A., in 2024 58th Asilomar Conference on Signals, Systems, and Computers. (2024), pp. 536–543.

[29] Schmuker M., Schneider G., Processing and classification of chemical data inspired by insect olfaction. Proceedings of the National Academy of Sciences 104, 20285–20289 (2007). 10.1073/pnas.0705683104 PMC2154423 18077325

[30] Snitz K. Predicting odor perceptual similarity from odor structure. PLoS computational biology 9, e1003184 (2013). 24068899 10.1371/journal.pcbi.1003184 PMC3772038

[31] Jiang Y. Molecular profiling of activated olfactory neurons identifies odorant receptors for odors in vivo. Nature neuroscience 18, 1446–1454 (2015). 26322927 10.1038/nn.4104 PMC4583814

[32] Vihani A. Encoding of odors by mammalian olfactory receptors. bioRxiv, 2021.2012. 2027.474279 (2021).

[33] Knight Zachary A. Molecular Profiling of Activated Neurons by Phosphorylated Ribosome Capture. Cell 151, 1126–1137 (2012). 23178128 10.1016/j.cell.2012.10.039 PMC3839252

[34] Mombaerts P., Odorant receptor gene choice in olfactory sensory neurons: the one receptor–one neuron hypothesis revisited. Current opinion in neurobiology 14, 31–36 (2004). 15018935 10.1016/j.conb.2004.01.014

[35] Serizawa S., Miyamichi K., Sakano H., One neuron-one receptor rule in the mouse olfactory system. Trends Genet 20, 648–653 (2004). 15522461 10.1016/j.tig.2004.09.006

[36] Chess A., Simon I., Cedar H., Axel R., Allelic inactivation regulates olfactory receptor gene expression. Cell 78, 823–834 (1994). 8087849 10.1016/s0092-8674(94)90562-2

[37] Malnic B., Hirono J., Sato T., Buck L. B., Combinatorial receptor codes for odors. Cell 96, 713–723 (1999). 10089886 10.1016/s0092-8674(00)80581-4

[38] Touhara K. Functional identification and reconstitution of an odorant receptor in single olfactory neurons. Proc Natl Acad Sci U S A 96, 4040–4045 (1999). 10097159 10.1073/pnas.96.7.4040 PMC22416

[39] Monahan K., Horta A., Lomvardas S., LHX2-and LDB1-mediated trans interactions regulate olfactory receptor choice. Nature 565, 448–453 (2019). 30626972 10.1038/s41586-018-0845-0 PMC6436840

[40] Shayya H. J. ER stress transforms random olfactory receptor choice into axon targeting precision. Cell 185, 3896–3912.e3822 (2022). 36167070 10.1016/j.cell.2022.08.025 PMC9588687

[41] Vihani A. Semiochemical responsive olfactory sensory neurons are sexually dimorphic and plastic. Elife 9, e54501 (2020). 33231170 10.7554/eLife.54501 PMC7732343

[42] Isogai Y. Multisensory logic of infant-directed aggression by males. Cell 175, 1827–1841. e1817 (2018). 30550786 10.1016/j.cell.2018.11.032 PMC6558521

[43] Ravia A. A measure of smell enables the creation of olfactory metamers. Nature 588, 118–123 (2020). 33177711 10.1038/s41586-020-2891-7

[44] Morgan H. L., The generation of a unique machine description for chemical structures-a technique developed at chemical abstracts service. Journal of chemical documentation 5, 107–113 (1965).

[45] Barsainyan A. A., Kumar R., Saha P., Schmuker M.. (2023).

[46] He H., Garcia E. A., Learning from Imbalanced Data. IEEE Transactions on Knowledge and Data Engineering 21, 1263–1284 (2009).

[47] Olender T., Jones T. E., Bruford E., Lancet D., A unified nomenclature for vertebrate olfactory receptors. BMC Evolutionary Biology 20, 42 (2020). 32295537 10.1186/s12862-020-01607-6 PMC7160942

[48] Yeung W. Tree visualizations of protein sequence embedding space enable improved functional clustering of diverse protein superfamilies. Briefings in Bioinformatics 24, (2023). 10.1093/bib/bbac619 PMC9851311 36642409

[49] Guerra J. V. d. S. pyKVFinder: an efficient and integrable Python package for biomolecular cavity detection and characterization in data science. BMC Bioinformatics 22, 607 (2021). 34930115 10.1186/s12859-021-04519-4 PMC8685811

[50] Richardson E. The receiver operating characteristic curve accurately assesses imbalanced datasets. Patterns 5, (2024). 10.1016/j.patter.2024.100994 PMC11240176 39005487

[51] Ijichi C. Metabolism of Odorant Molecules in Human Nasal/Oral Cavity Affects the Odorant Perception. Chem Senses 44, 465–481 (2019). 31254383 10.1093/chemse/bjz041

[52] Dobin A. STAR: ultrafast universal RNA-seq aligner. Bioinformatics 29, 15–21 (2013). 23104886 10.1093/bioinformatics/bts635 PMC3530905

[53] Li B., Dewey C. N., RSEM: accurate transcript quantification from RNA-Seq data with or without a reference genome. BMC bioinformatics 12, 323 (2011). 21816040 10.1186/1471-2105-12-323 PMC3163565

[54] Robinson M. D., McCarthy D. J., Smyth G. K., edgeR: a Bioconductor package for differential expression analysis of digital gene expression data. bioinformatics 26, 139–140 (2010). 19910308 10.1093/bioinformatics/btp616 PMC2796818

[55] Smedley D. BioMart–biological queries made easy. BMC genomics 10, 22 (2009). 19144180 10.1186/1471-2164-10-22 PMC2649164

[56] Hu X. S. Concentration-dependent recruitment of mammalian odorant receptors. eneuro 7, (2020). 10.1523/ENEURO.0103-19.2019 PMC7189481 32015097

[57] Rogers D., Hahn M., Extended-connectivity fingerprints. Journal of chemical information and modeling 50, 742–754 (2010). 20426451 10.1021/ci100050t

[58] Keller A., Vosshall L. B., Olfactory perception of chemically diverse molecules. BMC Neurosci 17, 55 (2016). 27502425 10.1186/s12868-016-0287-2 PMC4977894

[59] Katoh K., Kuma K.-i., Toh H., Miyata T., MAFFT version 5: improvement in accuracy of multiple sequence alignment. Nucleic acids research 33, 511–518 (2005). 15661851 10.1093/nar/gki198 PMC548345

[60] Darriba D. ModelTest-NG: a new and scalable tool for the selection of DNA and protein evolutionary models. Molecular biology and evolution 37, 291–294 (2020). 31432070 10.1093/molbev/msz189 PMC6984357

[61] Grantham R., Amino acid difference formula to help explain protein evolution. science 185, 862–864 (1974). 4843792 10.1126/science.185.4154.862

[62] Zhang Y., Skolnick J., TM-align: a protein structure alignment algorithm based on the TM-score. Nucleic acids research 33, 2302–2309 (2005). 15849316 10.1093/nar/gki524 PMC1084323

[63] Saito H., Kubota M., Roberts R. W., Chi Q., Matsunami H., RTP family members induce functional expression of mammalian odorant receptors. Cell 119, 679–691 (2004). 15550249 10.1016/j.cell.2004.11.021

[64] Zhuang H., Matsunami H., Evaluating cell-surface expression and measuring activation of mammalian odorant receptors in heterologous cells. Nat Protoc 3, 1402–1413 (2008). 18772867 10.1038/nprot.2008.120 PMC2664838

