# Supplementary material for: Decoding Smell from Receptor Structure
Source: Res Sq. 2026 Jun 14:rs.3.rs-9947738. Preprint. [Version 1] doi: 10.21203/rs.3.rs-9947738/v1 (PMC13278317; doi:10.21203/rs.3.rs-9947738/v1)
Supplement: 1 [file NIHPPrs9947738v1-supplement-1.pdf]

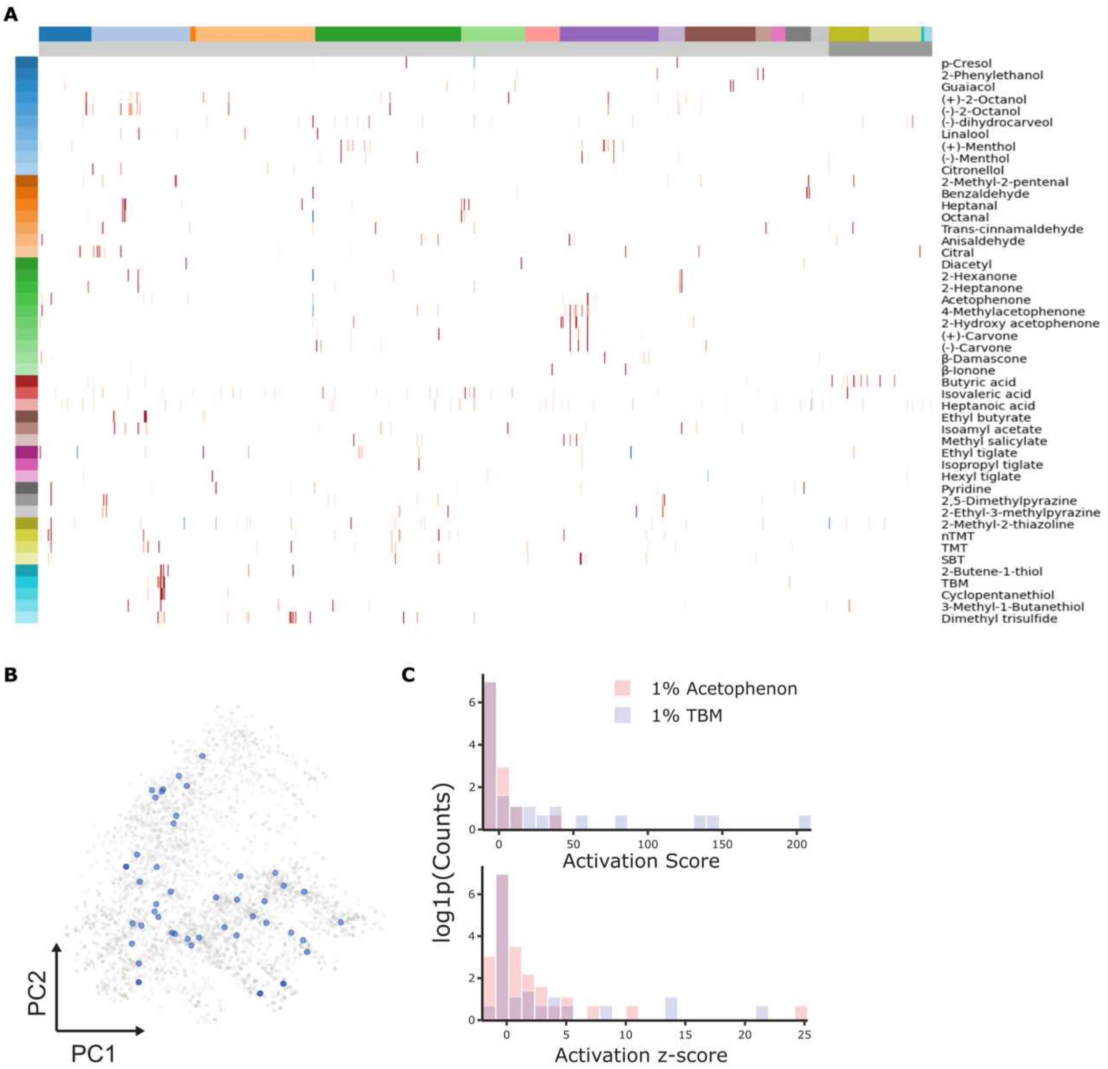

Figure S1. Global overview of pS6-IP activation profiles and chemical space coverage.

A. Heatmap of pS6-IP–derived activation z-scores across all tested odorant–receptor pairs. Rows correspond to the 48 odorants, and columns represent individual ORs, organized by class and family. **B.** Chemical space representation of odorants based on Morgan fingerprint principal component analysis (PCA). Odorants included in the pS6-IP panel are highlighted in blue relative to the full reference set.

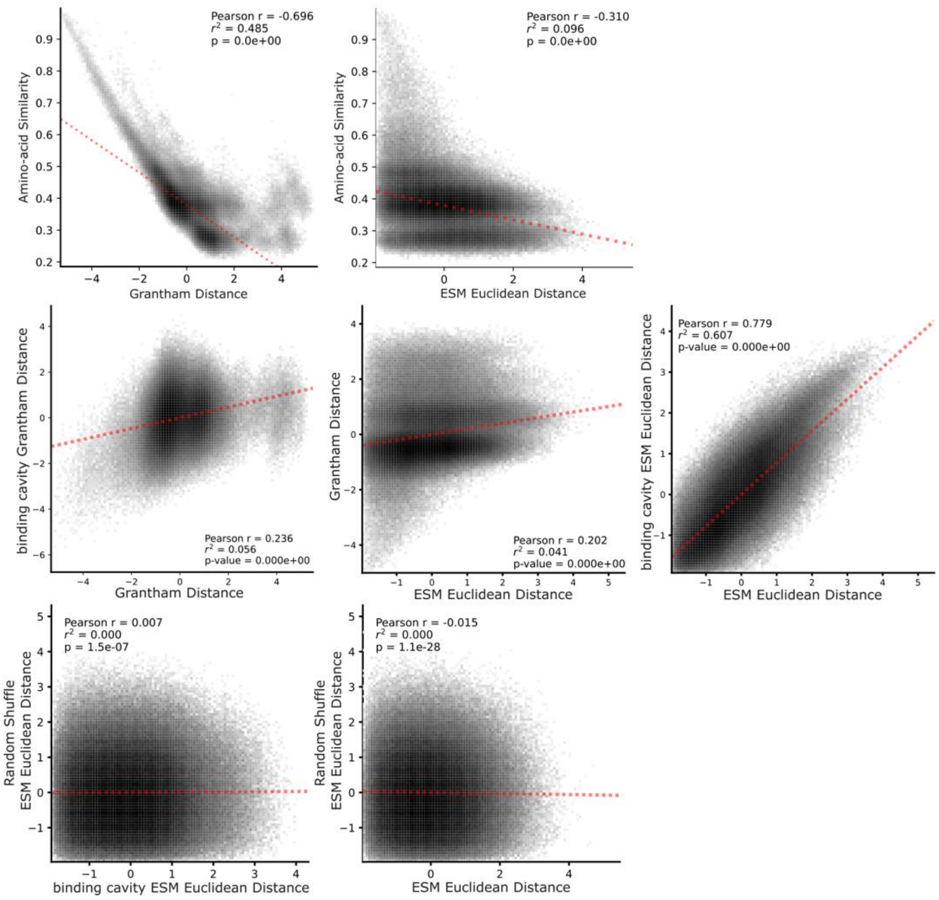

Figure S2. Comparison of sequence- and embedding-based similarity metrics across olfactory receptors.

Top: Pearson correlation analysis comparing amino acid similarity–based Grantham distances with ESM-derived Euclidean distances across OR pairs. Middle: Pairwise Pearson correlations among sequence- and structure-informed similarity measures, including full-length sequence Grantham distance vs. binding cavity Grantham distance, Grantham distance vs. ESM Euclidean distance, and binding cavity ESM Euclidean distance vs. full-sequence ESM Euclidean distance. Bottom: Control comparisons against randomized embeddings. Pearson

correlations between binding cavity ESM Euclidean distances and randomly shuffled ESM embeddings, and between full-sequence ESM Euclidean distances and shuffled controls, demonstrating preservation of biological signal in learned representations.

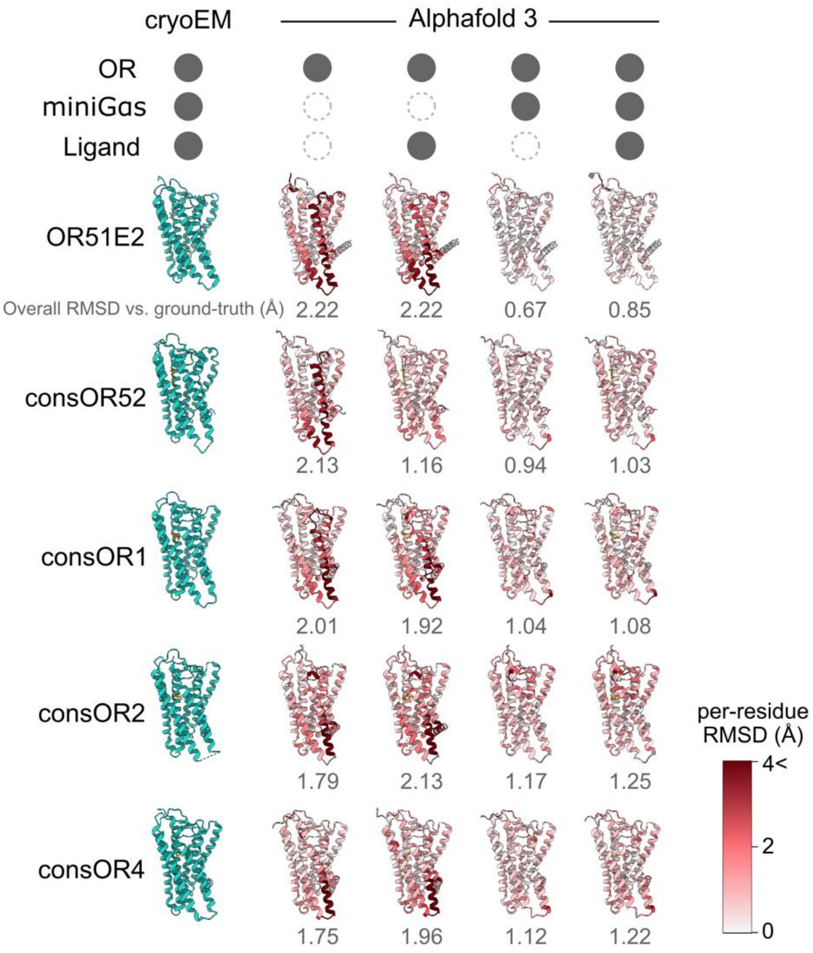

Figure S3. Structural agreement between cryo-EM olfactory receptor structures and AlphaFold3 predictions.

Comparison of experimentally determined cryo-EM structures with AlphaFold3-predicted models across multiple modeling conditions, including receptor alone (OR), receptor with ligand (OR + ligand), receptor with mini G protein (OR + miniGαs), and receptor with both ligand and miniGαs protein (OR + ligand + miniGαs). For each receptor, per-residue root-mean-

square deviation (RMSD) relative to the cryo-EM structure is shown to assess local structural agreement, alongside overall backbone RMSD values summarized below each model.

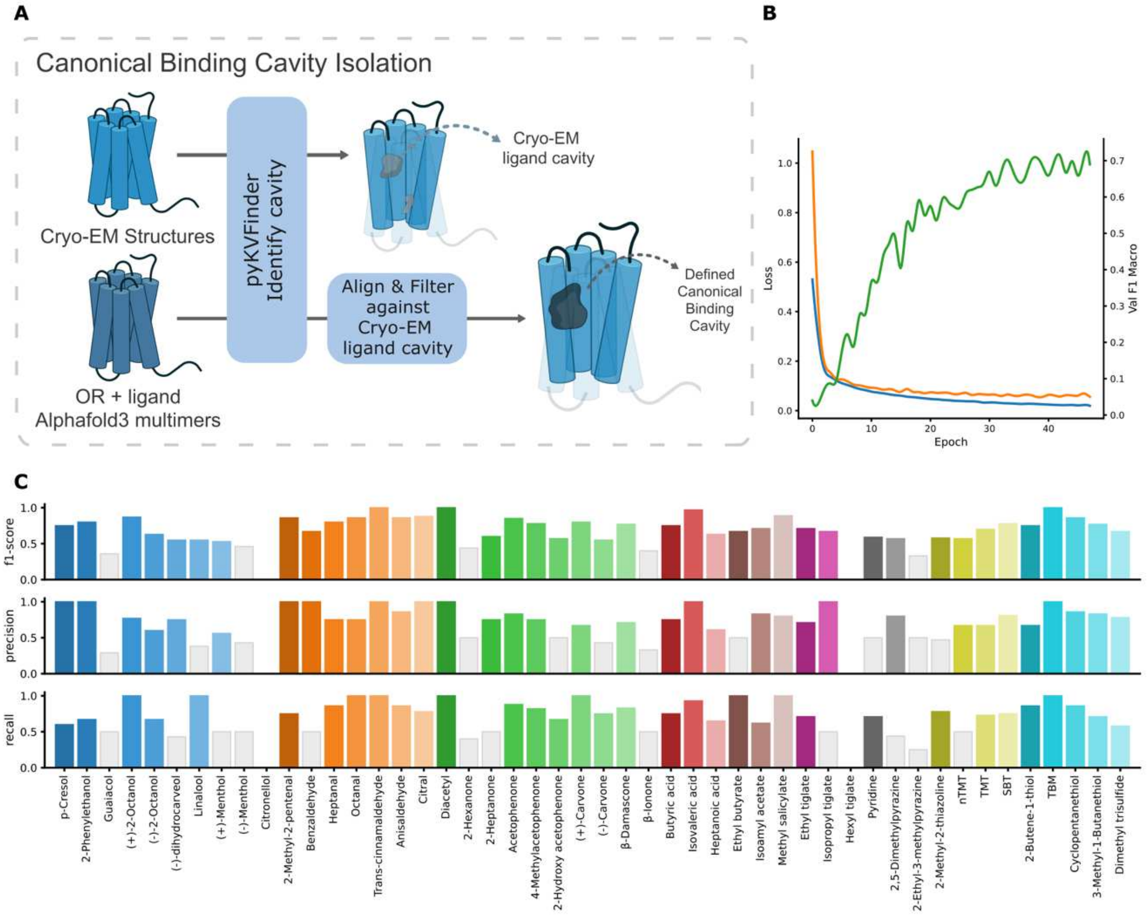

Figure S4. Binding cavity definition and model performance evaluation.

A. Schematic of the pipeline for canonical binding cavity identification. Cryo-EM structures and corresponding AlphaFold3-predicted models (OR + ligand + miniGαs) were analyzed using pyKVFinder to detect putative cavities. The cryo-EM derived cavity was used as a reference to align and superimpose predicted cavities. The resulting consensus region was defined as the canonical binding cavity used for downstream filtering and voxelization (see Methods for details). **B.** Training dynamics of the final CNN model. Loss curves for training (blue) and validation (orange) are shown across epochs, alongside validation macro F1-score

(green), demonstrating stable optimization and concordant performance improvement. **C.** Precision, recall, and F1-score for each of the 48 odorants. Odorants with performance values below 0.5 are highlighted in gray.

Figure

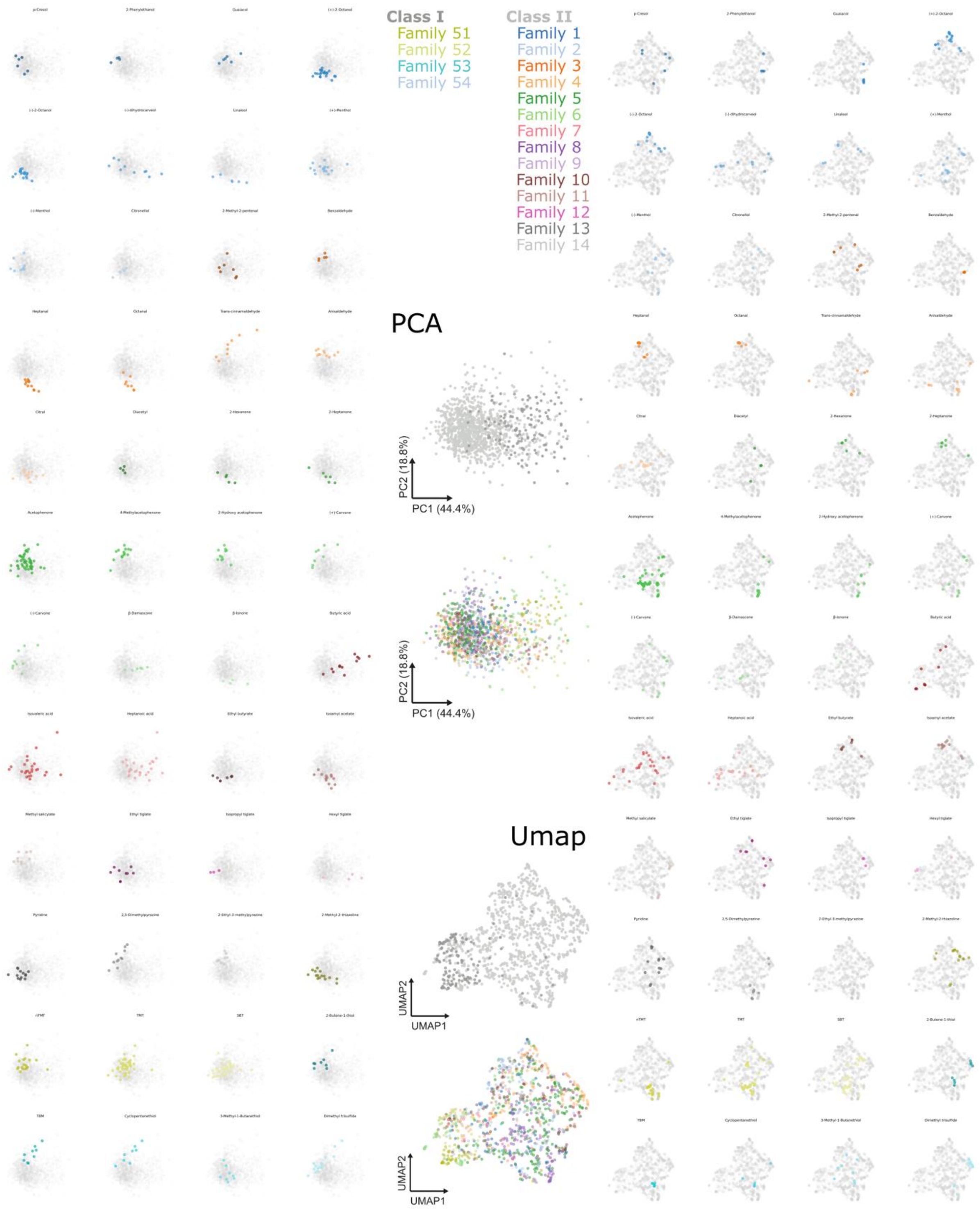
Class I
Family 51
Family 52
Family 53
Family 54
Class II
Family 1
Family 2
Family 3
Family 4
Family 5
Family 6
Family 7
Family 8
Family 9
Family 10
Family 11
Family 12
Family 13
Family 14
PCA
PC2 (18.8%)
PC1 (44.4%)
PC2 (18.8%)
PC1 (44.4%)
Umap
UMAP2
UMAP1
UMAP2
UMAP1

S5. Global organization of CNN-derived receptor embeddings across dimensionality reduction methods.

Comprehensive visualization of receptor embeddings using principal component analysis (PCA) and UMAP (smeLLMap). Left: PCA projections showing OR distributions for each of the 48 odorants, with receptors responding to each ligand highlighted. Right: Corresponding UMAP (smeLLMap) projections illustrating odor-specific clustering in the learned embedding space. Middle: Distribution of OR classes and families mapped onto both PCA and UMAP representations, demonstrating that higher-level class separation is preserved while finer family-level organization is less pronounced relative to functional clustering by odor response.

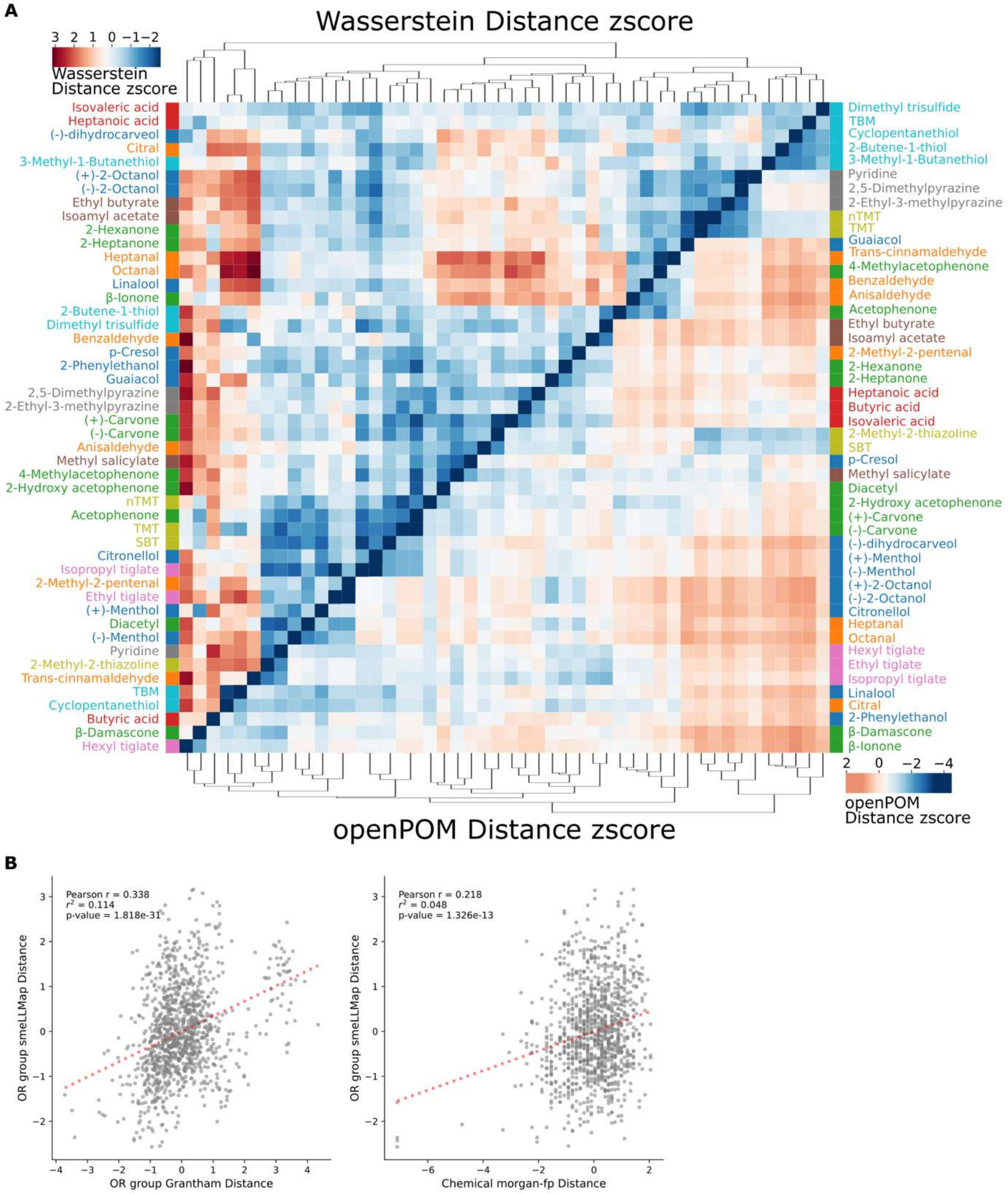
A
Wasserstein Distance zscore
3 2 1 0 −1 −2
Wasserstein Distance zscore
Isovaleric acid
Heptanoic acid
(-)-dihydrocarveol
Citral
3-Methyl-1-Butanethiol
(+)-2-Octanol
(-)-2-Octanol
Ethyl butyrate
Isoamyl acetate
2-Hexanone
2-Heptanone
Heptanal
Octanal
Linalool
β-Ionone
2-Butene-1-thiol
Dimethyl trisulfide
Benzaldehyde
p-Cresol
2-Phenylethanol
Guaiacol
2,5-Dimethylpyrazine
2-Ethyl-3-methylpyrazine
(+)-Carvone
(-)-Carvone
Anisaldehyde
Methyl salicylate
4-Methylacetophenone
2-Hydroxy acetophenone
nTMT
Acetophenone
TMT
SBT
Citronellol
Isopropyl tiglate
2-Methyl-2-pentenal
Ethyl tiglate
(+)-Menthol
Diacetyl
(-)-Menthol
Pyridine
2-Methyl-2-thiazoline
Trans-cinnamaldehyde
TBM
Cyclopentanethiol
Butyric acid
β-Damascone
Hexyl tiglate
Dimethyl trisulfide
TBM
Cyclopentanethiol
2-Butene-1-thiol
3-Methyl-1-Butanethiol
Pyridine
2,5-Dimethylpyrazine
2-Ethyl-3-methylpyrazine
nTMT
TMT
Guaiacol
Trans-cinnamaldehyde
4-Methylacetophenone
Benzaldehyde
Anisaldehyde
Acetophenone
Ethyl butyrate
Isoamyl acetate
2-Methyl-2-pentenal
2-Hexanone
2-Heptanone
Heptanoic acid
Butyric acid
Isovaleric acid
2-Methyl-2-thiazoline
SBT
p-Cresol
Methyl salicylate
Diacetyl
2-Hydroxy acetophenone
(+)-Carvone
(-)-Carvone
(-)-dihydrocarveol
(+)-Menthol
(-)-Menthol
(+)-2-Octanol
(-)-2-Octanol
Citronellol
Heptanal
Octanal
Hexyl tiglate
Ethyl tiglate
Isopropyl tiglate
Linalool
Citral
2-Phenylethanol
β-Damascone
β-Ionone
2 0 −2 −4
openPOM Distance zscore
openPOM Distance zscore
B
Pearson r = 0.338
r^2 = 0.114
p-value = 1.818e-31
OR group smeLLMap Distance
OR group Grantham Distance
Pearson r = 0.218
r^2 = 0.048
p-value = 1.326e-13
OR group smeLLMap Distance
Chemical morgan-fp Distance

Figure S6. Comparison of embedding-derived receptor distances with sequence- and chemistry-based similarity metrics.

A. Dual-clustered heatmaps comparing normalized distance metrics across odorants. Top: z-scored Wasserstein distances between distributions of OR embeddings (smeLLMap) for each odorant pair. Bottom: corresponding z-scored Euclidean distances in OpenPOM space, representing perception-informed chemical similarity. **B.** Correlation analyses comparing receptor embedding distances with alternative similarity measures. Left: Pearson correlation between OR group distances in smeLLMap (Wasserstein) and sequence-based Grantham distances. Right: Pearson correlation between OR group distances in smeLLMap and chemical similarity derived from Morgan fingerprint representations

	aucroc	prob_corr	f1-score	precision	recall
p-Cresol	0.89	0.73255	0.75	1	0.6
2-Phenylethanol	0.9422442	0.80710	0.8	1	0.67
Guaiacol	0.9751243	0.49389	0.36	0.29	0.5
(+)-2-Octanol	1	0.91053	0.87	0.77	1
(-)-2-Octanol	0.9620181	0.67341	0.63	0.6	0.67
(-)-dihydrocarveol	0.9639249	0.69319	0.55	0.75	0.43
Linalool	1	0.72351	0.55	0.38	1
(+)-Menthol	0.8558974	0.62406	0.53	0.56	0.5
(-)-Menthol	0.9480737	0.51978	0.46	0.43	0.5
Citronellol	0.9639303	0.37660	0	0	0
2-Methyl-2-pentenal	0.9726368	0.84515	0.86	1	0.75
Benzaldehyde	0.9827586	0.68817	0.67	1	0.5
Heptanal	0.9891774	0.73052	0.8	0.75	0.86
Octanal	1	0.90210	0.86	0.75	1
Trans-cinnamaldehyde	1	0.98596	1	1	1
Anisaldehyde	0.9963925	0.86971	0.86	0.86	0.86
Citral	0.9948979	0.86625	0.88	1	0.78
Diacetyl	1	0.89681	1	1	1
2-Hexanone	0.962	0.52969	0.44	0.5	0.4
2-Heptanone	0.9949748	0.75804	0.6	0.75	0.5
Acetophenone	0.9501409	0.81457	0.85	0.83	0.88
4-Methylacetophenone	0.9582942	0.83288	0.78	0.75	0.82
2-Hydroxy	0.9690117	0.62587	0.57	0.5	0.67
(+)-Carvone	1	0.90482	0.8	0.67	1
(-)-Carvone	0.9912935	0.70908	0.55	0.43	0.75
Œ≤-Damascone	0.9120603	0.86859	0.77	0.71	0.83
Œ≤-Ionone	0.9458128	0.49842	0.4	0.33	0.5
Butyric acid	0.9987562	0.88191	0.75	0.75	0.75
Isovaleric acid	0.9971929	0.91817	0.97	1	0.93
Heptanoic acid	0.8989361	0.66110	0.63	0.61	0.65
Ethyl butyrate	0.9926108	0.57559	0.67	0.5	1
Isoamyl acetate	0.9574873	0.74228	0.71	0.83	0.62
Methyl salicylate	1	0.90854	0.89	0.8	1
Ethyl tiglate	0.9855699	0.71354	0.71	0.71	0.71
Isopropyl tiglate	1	0.79596	0.67	1	0.5
Hexyl tiglate	0.9141791	0.05535	0	0	0
Pyridine	0.8593073	0.67125	0.59	0.5	0.71
2,5-Dimethylpyrazine	0.9739229	0.67666	0.57	0.8	0.44
2-Ethyl-3-	0.9315920	0.38868	0.33	0.5	0.25
2-Methyl-2-thiazoline	0.9387755	0.63984	0.58	0.47	0.78
nTMT	0.7512437	0.64863	0.57	0.67	0.5

TMT	0.9696969	0.76199	0.7	0.67	0.73
SBT	0.9461259	0.75225	0.78	0.81	0.75
2-Butene-1-thiol	0.8607503	0.78151	0.75	0.67	0.86
TBM	1	0.99962	1	1	1
Cyclopentanethiol	0.998557	0.92029	0.86	0.86	0.86
3-Methyl-1-Butanethiol	0.9812409	0.80131	0.77	0.83	0.71
Dimethyl trisulfide	0.9827288	0.68576	0.67	0.78	0.58

Table S1. Trained model's validation odor prediction metric

Summary of validation performance for each odorant, including area under the receiver operating characteristic curve (AUROC), probability–response correlation (prob_corr), F1-score, precision, and recall.

	odor	cid	n_OR	obs_grantham_mean	obs_grantham_median	null_grantham_mean	null_grantham_median	p_grantham_perm_mean	p_grantham_perm_median	obs_euclid_mean	obs_euclid_median	null_euclid_mean	null_euclid_median	p_euclid_perm_mean	p_euclid_perm_median
0	Dimethyl trisulfide	19310	16	0.014	0.251	0.005	0.037	0.502	0.826	-0.443	-0.915	0.012	-0.163	0.056	0
1	3-Methyl-1-Butanethiol	10925	8	-0.600	-0.642	-0.005	0.022	0.036	0.032	-0.581	-0.556	-0.014	-0.151	0.06	0.132
2	Cyclopentanethiol	15510	9	-1.254	-0.716	0.007	0.032	0	0.008	-0.283	-0.444	0.040	-0.108	0.198	0.178
3	TBM	6387	10	-1.371	-0.836	-0.012	0.027	0	0.002	-0.703	-0.789	0.020	-0.141	0.018	0.014
4	2-Butene-1-thiol	6433451	9	-0.128	-0.056	-0.022	0.014	0.374	0.424	-0.764	-0.769	0.024	-0.126	0.016	0.028
5	SBT	162148	49	-0.331	-0.297	-0.004	0.022	0	0	-0.752	-0.866	0.007	-0.158	0	0
6	TMT	263626	45	-0.358	-0.284	-0.003	0.013	0.002	0.014	-0.661	-0.813	0.014	-0.158	0	0
7	nTMT	61653	19	-0.610	-0.602	-0.006	-0.002	0	0	-0.573	-0.627	0.009	-0.156	0.014	0.024
8	2-Methyl-2-thiazoline	16867	15	0.131	0.200	0.006	0.027	0.702	0.778	-0.725	-0.907	-0.014	-0.184	0.004	0
9	2-Ethyl-3-methylpyrazine	27457	9	-0.191	0.155	-0.003	0.001	0.252	0.684	-0.953	-0.898	0.009	-0.123	0.006	0.016
10	2,5-Dimethylpyrazine	31252	11	-1.021	-0.758	0.005	0.043	0	0.004	-0.655	-0.714	0.000	-0.187	0.016	0.022
11	Pyridine	1049	13	-0.169	-0.076	0.003	0.020	0.228	0.322	-0.944	-1.028	0.019	-0.138	0	0
12	Hexyl tiglate	637523	4	1.175	1.071	0.003	0.045	0.992	0.974	0.064	-0.064	0.003	-0.099	0.584	0.522
13	Isopropyl tiglate	5367745	3	-0.800	-0.571	0.019	0.070	0.114	0.196	-1.421	-1.464			0.012	0.01
14	Ethyl tiglate	5281163	10	-0.234	0.106	-0.007	0.006	0.248	0.602	-0.572	-0.549	-0.033	-0.165	0.08	0.152
15	Methyl salicylate	4133	9	-0.503	-0.477	-0.013	0.000	0.036	0.042	-1.018	-1.061	0.007	-0.146	0.002	0.002
16	Isoamyl acetate	31276	15	-0.612	-0.717	-0.003	0.029	0	0	-0.930	-0.982	0.011	-0.135	0	0
17	Ethyl butyrate	7762	9	-1.008	-0.419	0.005	0.006	0	0.086	-0.926	-0.575	-0.003	-0.148	0.004	0.09
18	Heptanoic acid	8094	29	-0.059	-0.013	0.001	0.021	0.35	0.41	-0.170	-0.324	0.015	-0.158	0.172	0.186
19	Isovaleric acid	10430	25	0.069	0.072	-0.004	0.009	0.666	0.638	-0.142	-0.440	-0.001	-0.164	0.26	0.078
20	Butyric acid	264	13	-0.843	-0.516	0.000	0.039	0	0.014	0.032	-0.185	0.010	-0.157	0.52	0.468
21	Œ≤-Ionone	638014	4	-0.278	-0.356	0.047	0.110	0.264	0.226	-0.435	-0.693	0.002	-0.091	0.256	0.122
22	Œ≤-Damascone	32052	6	-0.271	-0.116	-0.017	0.043	0.262	0.366	-0.863	-0.865	-0.010	-0.149	0.032	0.036
23	(-)-Carvone	439570	9	-0.910	-0.622	0.013	0.044	0.002	0.022	-0.259	-0.320	0.011	-0.137	0.256	0.312
24	(+)-Carvone	16724	8	-1.013	-0.816	-0.017	0.008	0.004	0.016	-0.397	-0.601	-0.021	-0.171	0.19	0.12
25	2-Hydroxy acetophenone	68490	11	-1.247	-0.796	-0.004	0.040	0	0.006	-1.050	-1.164	-0.016	-0.159	0	0
26	4-Methylacetophenone	8500	14	-1.515	-1.152	-0.004	0.031	0	0	-0.967	-1.086	0.033	-0.124	0	0
27	Acetophenone	7410	50	-0.409	-0.358	-0.006	0.019	0	0	-0.662	-0.706	-0.011	-0.174	0	0
28	2-Heptanone	8051	6	-1.072	-1.058	-0.006	0.015	0.006	0.006	-0.735	-1.090	0.010	-0.168	0.048	0.004
29	2-Hexanone	11583	5	-1.506	-1.657	0.013	0.061	0	0	-0.971	-0.958	0.060	-0.077	0.012	0.02
30	Diacetyl	650	4	-0.280	-0.176	-0.013	0.025	0.334	0.382	-1.494	-1.543	-0.002	-0.071	0	0
31	Citral	638011	12	-0.847	-0.837	-0.007	0.021	0	0	-0.914	-1.021	-0.010	-0.172	0	0
32	Anisaldehyde	31244	11	-0.731	-0.282	-0.017	0.007	0.01	0.144	-1.019	-1.069	0.035	-0.117	0.002	0
33	Trans-cinnamaldehyde	637511	8	0.138	0.211	0.012	0.012	0.67	0.696	0.126	-0.104	0.001	-0.127	0.642	0.528
34	Octanal	454	8	-1.601	-1.352	0.000	0.024	0	0	-1.175	-1.100	-0.016	-0.165	0	0.002
35	Heptanal	8130	11	-0.968	-0.877	0.009	0.024	0	0.002	-1.289	-1.330	0.001	-0.167	0	0
36	Benzaldehyde	240	5	0.230	-0.043	0.008	0.023	0.698	0.46	-1.492	-1.387	0.022	-0.123	0	0
37	2-Methyl-2-pentenal	5319754	7	-0.433	-0.089	-0.001	-0.001	0.1	0.39	-0.462	-0.436	0.006	-0.133	0.148	0.25
38	Citronellol	8842	5	-0.415	0.131	-0.009	0.012	0.172	0.6	-1.160	-1.309	-0.001	-0.135	0.006	0
39	(-)-Menthol	16666	9	-0.581	-0.668	-0.021	0.006	0.04	0.024	-0.924	-0.945	-0.023	-0.171	0.006	0.012
40	(+)-Menthol	165675	15	-0.935	-0.844	-0.005	0.017	0	0	-0.659	-0.730	0.008	-0.175	0.002	0.006
41	Linalool	6549	6	-0.929	-0.825	-0.006	0.014	0.012	0.038	-0.453	-0.353	0.026	-0.099	0.146	0.28
42	(-)-dihydrocarveol	443163	10	-0.201	-0.126	0.016	0.048	0.232	0.316	0.527	0.217	-0.002	-0.170	0.92	0.834
43	(-)-2-Octanol	80080	17	-0.822	-0.855	-0.004	0.028	0	0	-1.124	-1.114	0.006	-0.165	0	0
44	(+)-2-Octanol	2723888	22	-0.280	-0.305	0.000	0.032	0.068	0.064	-0.974	-1.079	0.013	-0.157	0	0
45	Guaiacol	460	6	-0.864	-0.334	-0.014	0.021	0.014	0.186	-1.024	-1.223	-0.011	-0.142	0.004	0.002
46	2-Phenylethanol	6054	4	-1.095	-0.968	-0.057	0.007	0.02	0.042	-1.258	-1.341	0.020	-0.026	0.002	0
47	p-Cresol	2879	6	-0.806	-0.787	-0.024	0.024	0.026	0.048	-0.414	-0.385	-0.001	-0.111	0.206	0.276

Table S2. Pairwise receptor similarity metrics for odor-specific responding ORs.

Summary of observed and permuted pairwise distances among ORs responding to each odorant, computed using Grantham sequence distances and smeLLMap embedding Euclidean distances. For each odorant, mean and median distances are reported alongside corresponding null distributions generated by random permutation, with associated permutation-based *p-values* indicating statistical significance.
